# Serum Proteomic Changes after Randomized Prolonged Erythropoietin Treatment and/or Endurance Training: Detection of Novel Biomarkers

**DOI:** 10.1371/journal.pone.0117119

**Published:** 2015-02-13

**Authors:** Britt Christensen, Maja Ludvigsen, Birgitte Nellemann, John J. Kopchick, Bent Honoré, Jens Otto L. Jørgensen

**Affiliations:** 1 Department of Endocrinology and Internal Medicine, Aarhus University Hospital, Aarhus, Denmark; 2 Medical Research Laboratories, Aarhus University, Aarhus, Denmark; 3 Department of Biomedicine, Aarhus University, Aarhus, Denmark; 4 Edison Biotechnology Institute Ohio University, Athens, Ohio, United States of America; 5 Department of Biomedical Sciences, Heritage College of Osteopathic Medicine, Ohio University, Athens, Ohio, United States of America; 6 Research Laboratory for Biochemical Pathology, Institute for Clinical Medicine, Aarhus University Hospital, Aarhus, Denmark; Vanderbilt University, UNITED STATES

## Abstract

**Introduction:**

Despite implementation of the biological passport to detect erythropoietin abuse, a need for additional biomarkers remains. We used a proteomic approach to identify novel serum biomarkers of prolonged erythropoiesis-stimulating agent (ESA) exposure (Darbepoietin-α) and/or aerobic training.

**Trial Design:**

Thirty-six healthy young males were randomly assigned to the following groups: Sedentary-placebo (n = 9), Sedentary-ESA (n = 9), Training-placebo (n = 10), or Training-ESA (n = 8). They were treated with placebo/Darbepoietin-α subcutaneously once/week for 10 weeks followed by a 3-week washout period. Training consisted of supervised biking 3/week for 13 weeks at the highest possible intensity. Serum was collected at baseline, week 3 (high dose Darbepoietin-α), week 10 (reduced dose Darbepoietin-α), and after a 3-week washout period.

**Methods:**

Serum proteins were separated according to charge and molecular mass (2D-gel electrophoresis). The identity of proteins from spots exhibiting altered intensity was determined by mass spectrometry.

**Results:**

Six protein spots changed in response to Darbepoietin-α treatment. Comparing all 4 experimental groups, two protein spots (serotransferrin and haptoglobin/haptoglobin related protein) showed a significant response to Darbepoietin-α treatment. The haptoglobin/haptoglobin related protein spot showed a significantly lower intensity in all subjects in the training-ESA group during the treatment period and increased during the washout period.

**Conclusion:**

An isoform of haptoglobin/haptoglobin related protein could be a new anti-doping marker and merits further research.

**Trial Registration:**

ClinicalTrials.gov NCT01320449

## Introduction

The production of red blood cells was linked to blood oxygen pressure more than a century ago [1]. In 1906 Carnot and DeFlandre described a substance capable of stimulating the production of red blood cells [2], later known as erythropoietin (Epo). Recombinant human erythropoietin (rHuEpo) was approved for treatment of chronic anemia mainly in patients with end-stage renal disease in 1989. Soon after in 1990, the American Medical Association and the International Olympic Committee banned the abuse of rHuEpo in athletes. However, abuse of rHuEpo and related compounds prevails.

In 2000 a direct method was developed to optimize and increase the sensitivity and specificity of detecting rHuEpo abuse. With this assay it is possible to distinguish endogenous Epo from rHuEpo in urine due to differences in glycosylation patterns, but with a limited window of detection [3]. The hematological passport was therefore implemented in 2009, which compares the individual´s measured blood values over time. Different markers are incorporated in the passport, but new markers are sought in order to optimize and increase the specificity of this indirect test [4]. The biomarkers in the blood passport have the advantage of being sensitive to present and future erythropoiesis-stimulating agents (ESAs) as well as gene doping, whereas a ‘direct’ test has to be established for every new drug [5].

The proteomic approach is a promising method for identification of novel anti-doping markers [6]. A potential strength of the gel-based proteomic approach is that numerous isoforms of a protein usually are detected, not only the total protein levels [7]. Isoforms of a protein are formed by post-translational modifications (PTM) which induce mass and/or charge shifts, such as protein cleavage and side-chain residue modifications [8]. Additionally, these modifications may change the activity of the protein. Thus, proteomic analyses make it possible to identify an isoform of a protein that responds specifically to a given treatment.

Previously, it has been shown that 16 days of rHuEpo administration induced significant changes in the isoforms of three serum proteins: haptoglobin, transferrin, and hemopexin [9]. However, it is important to know how these biomarkers respond to external factors such as physical activity and sojourn to altitude, all used by athletes frequently, before implementing these biomarkers in the hematological passport. It is also relevant to obtain detailed knowledge of the time course changes in these biomarkers relative to timing of Epo treatment. Thus, the training induced changes in the proteome as well as the window of detection for these biomarkers still needs to be determined.

In the present randomized and placebo-controlled study we therefore examined the serum proteome before, during, and after prolonged ESA (Darbepoietin-α) administration alone and in combination with a structured exercise program.

## Methods

### Ethics statement

Thirty-eight healthy, young, untrained men were included after providing a written informed consent to participate in adherence to the declaration of Helsinki. The study was approved by the Local Human Ethical Committee of Central Denmark Region (M-20110035), and registered at Clinical Trials (NCT01320449). The study and data analyses were performed at Aarhus University Hospital and Aarhus University, Denmark. The protocol for this trial and supporting CONSORT checklist are available as supporting information; see S1 CONSORT Checklist, S1 Protocol, and S2 Protocol. Results regarding metabolic adaptations [10] and effects on skeletal muscle tissue [11, 12] have been published.

### Subjects

Maximal oxygen uptake (VO_2_max) was measured prior to study start and only subjects with an oxygen uptake <50 ml/min/kg were included (moderate to low oxygen uptake). In addition, each participant underwent a physical examination and electrocardiogram measurements. Additional inclusion criteria included: age between 18–35 years, body mass index (BMI) between 18–29 kg/m^2^, normal blood pressure <135/85 mmHg, and a hematocrit <45%. Subjects were included between June 2011 and January 2012.

### Experimental design

**Fig 1:**
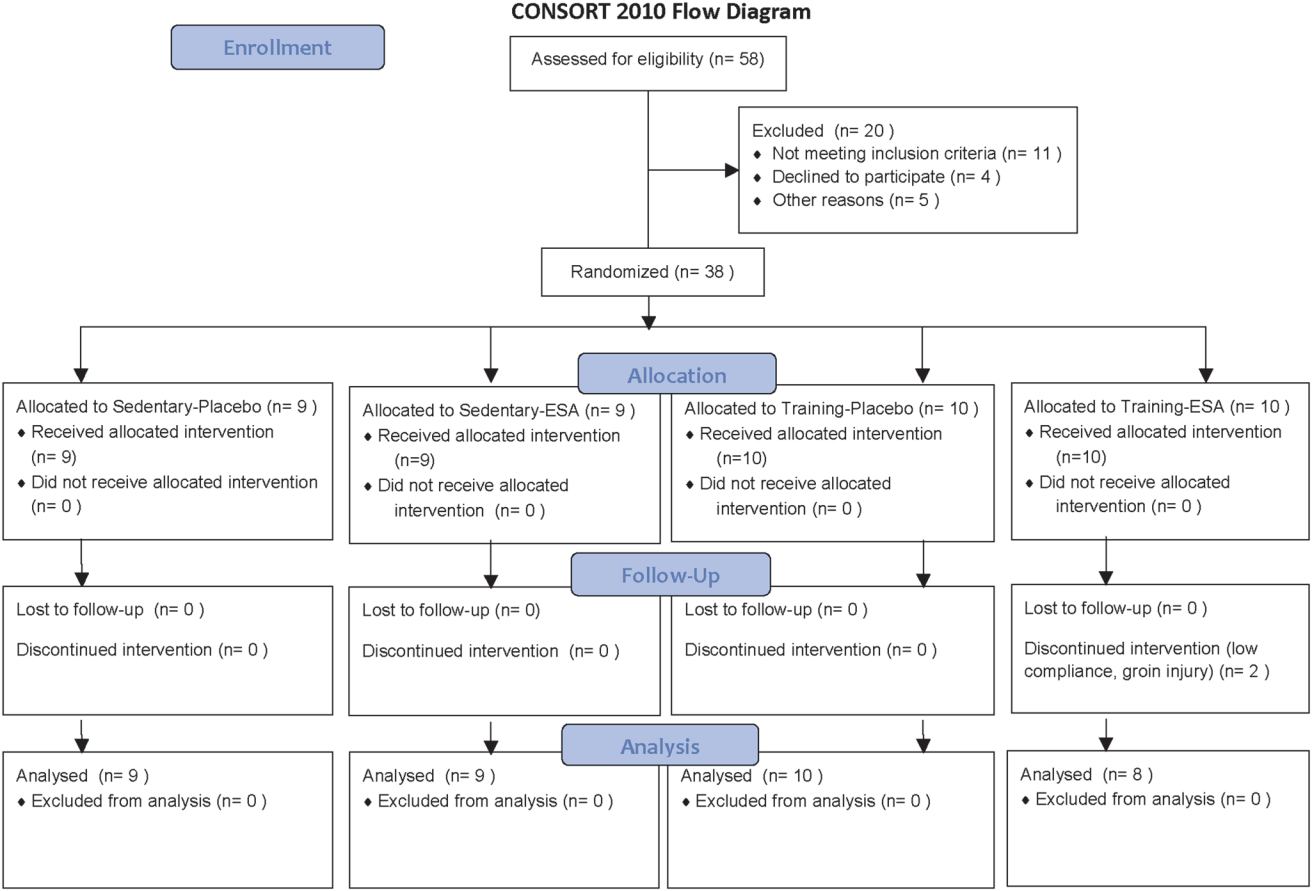
CONSORT flow diagram.

Our primary study outcome was the determination of biomarkers that responded to Darbepoietin-α treatment, but was not affected by endurance training. The study had a single-blinded, randomized design, and the participants were randomly assigned to one of four experimental groups; 1) placebo treatment (n = 9), 2) Darbepoietin-α treatment (n = 9), 3) endurance training (n = 10), and 4) Darbepoietin-α treatment and endurance training (n = 10) (Table 1, CONSORT flow diagram Fig. 1). Randomization was performed by subjects drawing an envelope; only the principal investigator where aware of the randomization. The sample size were chosen based on our previous study showing significantly altered serum proteome after 16 days of rHuEpo treatment in eight subjects, a few extra subjects were included due to the risk of dropout. Two subjects were omitted from the training-ESA group, one due to low compliance and one due to a groin injury (n = 8). The participants, lab technicians, and training instructors were blinded to the treatment; only the principal investigator and the physician providing the injections were not blinded. The participants were treated subcutaneously with either placebo or Darbepoietin-α (Aranesp, Amgen) for 10 weeks. Darbepoietin-α was administrated once weekly at a dose of 40 μg for three weeks, followed by 20 μg for the remaining seven weeks. However, the first three and four in the ESA group and training-ESA group, respectively, were treated twice weekly for the first three weeks. This resulted in a larger increase in hematocrit than expected, and the number of injections was, therefore, reduced. In order to keep the hematocrit values below 55%, some of the subjects (n = 9) were given placebo injections on a few occasions; the aim was to reach a hematocrit of ~50%. Darbepoietin-α has two extra oligosaccharide chains attached, which increases its half-life compared to endogenous Epo. All subjects were supplemented with 100 mg iron orally/day (Ferrous sulfate, Ferro Duretter, GlaxoSmithKline) from one week before the treatment and throughout the study period in order to prevent iron deficiency.

**Table 1:** Anthropometric data.

**Anthropometric data**					
	Sedentary-Placebo (n = 9)	Sedentary-ESA (n = 9)	Training-Placebo (n = 10)	Training-ESA (n = 8)	Interaction p-value
Age (years)	25.2 ± 1.6	22.7 ±1.0	20.9 ± 0.6 #	25.3 ± 1.7	0.046*
Weight (kg)	79.2 ± 3.3	82.1 ± 3.4	74.2 ± 2.7	78.8 ± 5.9	0.519
Height (m)	1.82 ± 0.02	1.87 ± 0.02	1.83 ± 0.02	1.81 ± 0.04	0.359
VO_2_max (L/min)	3.37 ± 0.20	3.56 ± 0.13	3.28 ± 0.18	3.05 ± 0.2	0.273
Erythrocytes (EVF)	42.8 ± 0.7	42.1 ±0.5	41.8 ± 0.6	42.1 ± 0.9	0.756

Data are expressed as mean ± SE. Significant interaction

* p<0.05, different from sedentary-placebo and training-ESA # p<0.05. EVF; erythrocyte volume fraction.

Serum samples were drawn before treatment/training were started (t = 0), after the boosting period (t = 3 weeks), after the treatment period (t = 10 weeks), and after a 3-week washout period (t = 13 weeks). Serum samples were centrifuged after collection (10 min, 4°C, 3,800 rpm) and stored at -20°C.

The endurance training consisted of indoor biking on an ergometer 3/week for 13 weeks. All training sessions were supervised, and the following three exercise sets were performed once weekly; 1) 40 min, 2) 2x20 min (5 min rest), and 3) 8x5 min (1 min rest), all sets were performed with the maximal amount of watt possible. Target workloads for the first three training sessions were calculated based on VO_2_ after the 5 min warm-up at 140 watt and VO_2_max. The aim was to work at 70% of VO_2_max (~58% wattmax) during the continual workout, at 80–90% VO_2_max (~70% wattmax) during the long intervals, and at 90–100% VO_2_max (~78% wattmax) during the short intervals; i.e target watt for a continual session was calculated as (70% * VO_2_max)/(VO_2_ at 140 watt/140 watt). Due to the non-predictable influence of Darbepoietin-α on performance it was deemed impracticable to determine a fixed progression in training intensity (Watts; W). Thus, to ensure equal conditions and a sufficient training intensity in the two training groups, the training instructors (who were blinded for the treatment intervention) determined target workloads for each session and aimed at increasing the absolute intensity and magnitude of work performed every week. All training was supervised and the mean workload performed during each session was recorded. To further assess training progress and to aid targeting of absolute training intensities, Wattmax-tests were incorporated into the training schedule by replacing an exercise session at week four and eight.

### Proteomic analysis

The procedures used for proteomic analysis have been described previously [13]. Serum samples were depleted of albumin and IgG. Proteins were first separated in the first dimension (1DE) according to isoelectric point followed by separation in the second dimension (2DE) according to mass. Proteins were stained by Sypro-Ruby and visualized by ImageQuant. Gel pictures were imported into and analyzed with PDQuest, which assigned a volume to each spot proportional to the amount of protein. The spots were matched between all gels, and spot intensity quantified. Spots that showed a significantly different expression were cut out of the gel.

### Protein identification by LC-MS/MS

Mass spectrometry analysis was performed as previously described [13]. Proteins were in-gel trypsinized overnight, pretreated with acetonitrile, reduced with DTT, blocked with iodoactemide, cleaved by trypsin, and the peptides were extracted by 1 change of Na_4_HCO_3_ and 3 changes of formic acid in acetonitrile. The extracted proteins were dried and then dissolved in 12 μl of buffer A (97.7% H_2_0, 2% acetonitrile, and 0.3% formic acid). Peptide separation before MS/MS analysis was performed using an Easy nLC II (Thermo Scientific). The samples were then analyzed on a Q-TOF Premier mass spectrometer (Waters). [Glu^1^]-fibrinopeptide B (GFP), 300 fmol/μl was used as lock mass injected at a flow rate of 300 nl/min. Calibration was also performed with GFP. MS survey scans were obtained using MassLynx v4.0 in the m/z interval between 450–1500 while MS/MS scans were obtained in the m/z interval between 50–1800. Raw data were processed using ProteinLynx Global Server v2.3. The processed data were used to search the Swiss-Prot Database using the online version of the Mascot MS/MS Ion Search facilities (Matrix Science, Ltd., http://www.matrixscience.com) [14]. Searching was performed with doubly, triply, and quadruply charged ions with up to two missed cleavages, a peptide mass tolerance of 20 ppm, and an MS/MS tolerance of 0.05 Da, one fixed modification, Carbamidomethyl (C), and variable modifications, oxidation (HWM) or dioxidation (M). Contaminating peptides and cross-contaminating peptides from previous samples including keratins, trypsin, and BSA were disregarded. Spectra of dubious identifications were manually evaluated and omitted if found to be of insufficient quality. At least one significant peptide (p<0.05) was required in order to qualify as a protein hit.

### Blood analysis

Hemoglobin, erythrocytes, and reticulocytes were measured on a Sysmex XE5000 (Sysmex, Ballerup, Denmark). Iron, iron saturation, haptoglobin, ferritin, and transferrin were measured on a Cobas 6000 (Roche Applied Sciences, Basel, Switzerland)

### Statistics

The level of significance was p<0.05. One-way ANOVA or three-way ANOVA with repeated measurements assessed differences between groups and/or over time. Due to a large biological variation between subjects, spot intensities were normalized to baseline (week 0) levels. Results are presented as mean ± SE (normally distributed) and as estimated medians and 95% confidence interval (CI95%) (non-normally distributed). Statistical analyses and graphical presentations were performed in STATA version 12 and SigmaPlot version 11.0.

## Results

All analyses were performed on all subjects.

### Aerobic capacity

Endurance training and Darbepoietin-α treatment for 10 weeks both significantly increased VO_2_max (time effect, p<0.001; time*training, p = 0.007; time*ESA, p = 0.003) [Sedentary-placebo: 3.37±0.20 to 3.41±0.23; Sedentary-ESA: 3.56±0.13 to 4.10±0.23; Training-placebo: 3.28±0.18 to 3.86±0.13; Training-ESA: 3.05±0.20 to 3.82±0.22 L/min], at baseline no difference between groups was found. Maximal workloads (watt_max_) significantly increased after 10 weeks of endurance training (time effect, p<0.001; time*training effect, p = 0.028) [Sedentary-placebo: 316±014 to 317±17; Sedentary-ESA: 325±10 to 350±15; Training-placebo: 316±15 to 369±9; Training-ESA: 303±12 to 364±13 watt] [10, 12].

### Hematological measurements

Changes in hematological variables are shown in Table 2. In short, Darbepoietin-α treatments lead to significant increases in reticulocytes (ret%) and erythrocytes (EVF) (ESA effect p = 0.020 and p<0.001, respectively). Although the subjects were given iron supplementation, Darbepoietin-α treatments lead to a small but significant decrease in iron levels within the normal range (ESA effect p = 0.015). Red blood cell distribution width (RDW) and mean corpuscular volume (MCV) significantly increased during the first weeks of Darbepoietin-α treatment (ESA effect p = 0.001 and ESA*time effect p = 0.004, respectively). On the other hand, a small decrease in mean corpuscular hemoglobin concentration (MCHC) was found during the first weeks after Darbepoietin-α treatment (ESA effect p = 0.007). An overall interaction between Darbepoietin-α and training was found for mean corpuscular hemoglobin (MCH) (ESA*training p = 0.020) with a significant change over time (time effect p = 0.005). Both transferrin and ferritin changed significantly over time (time effect p<0.001) and a significant interaction between time and Darbepoietin-α was found (ESA*time effect p<0.001).

**Table 2:** Hematological measurements.

**Hematological measurements**					
		Baseline	Week 3	Week 10	Week 13
Hemoglobin (g/dl)	SP	14.7 ± 0.2	15.3 ± 0.3 ^a^	14.6 ± 0.2 ^c^	15.5 ± 0.2 ^a^
	SE	14.4 ± 0.2	16.3 ± 0.3 ^a b e f^	16.0 ± 0.1 ^a^ *^b f^*	15.8 ± 0.2 ^a f^
	TP	14.7 ± 0.3	14.9 ± 0.3	14.4 ± 0.3 ^c^	15.1 ± 0.3
	TE	14.4 ± 0.3	16.2 ±0.2 ^a b f^	16.4 ± 0.2 ^a^ *^b f^*	16.1 ± 0.1 ^a f^
Hemoglobin (MCHC) (mmol/l)	SP	21.4 ± 0.2	21.4 ± 0.1 ^d^	21.6 ± 0.1	21.5 ± 0.1
	SE	21.3 ± 0.2 ^f^	21.1 ± 0.2	21.1 ± 0.1 ^b f^	21.3 ± 0.1
	TP	21.8 ± 0.2 ^b^	21.5 ± 0.1 ^a d^	21.7 ± 0.2	21.6 ± 0.2 ^a^
	TE	21.3 ± 0.1 ^f^	20.9 ± 0.1 ^a b d e f^	21.3 ± 0.1	21.3 ± 0.1
Hemoglobin (MCH) (fmol/cell)	SP	1.82 ± 0.01	1.84 ± 0.01	1.84 ± 0.02	1.84 ± 0.02
	SE	1.86 ± 0.01	1.88 ± 0.02 ^a b^	1.87 ± 0.02	1.86 ± 0.02
	TP	1.87 ± 0.02	1.87 ± 0.02 ^d^	1.90 ± 0.02 ^a b g^	1.88 ± 0.02 ^g^
	TE	1.82 ± 0.02	1.84 ± 0.02 ^a e^	1.84 ± 0.02	1.82 ± 0.02
Erythrocytes (EVF)	SP	42.8 ± 0.7	44.4 ± 0.8 ^a^	41.8 ± 0.5 ^c^	44.7 ± 0.8 ^a^
	SE	42.1 ± 0.5	48.1 ± 0.9 ^a b e f^	47.2 ± 0.5 ^a b e f^	46.0 ± 0.5 ^a f^
	TP	41.8 ± 0.6	43.3 ± 0.8 ^a^	41.4 ± 0.5 ^c^	43.4 ± 0.8 ^a^
	TE	42.1 ± 0.9	48.4 ± 0.6 ^a b f^	47.8 ± 0.6 ^a^ *^b f^*	47.1 ± 0.4 ^a b f^
Erythrocytes (10^12/l)	SP	5.0 ± 0.1	5.2 ± 0.1 ^a^	4.9 ± 0.1 ^c^	5.2 ± 0.1 ^a^
	SE	4.8 ± 0.1	5.4 ± 0.1 ^a f^	5.3 ± 0.1 ^a b f g^	5.3 ± 0.1 ^a f g^
	TP	4.9 ± 0.1	4.9 ± 0.1 ^b^	4.7 ± 0.1 ^a b^	5.0 ± 0.1 ^b d^
	TE	4.9 ± 0.1	5.5 ± 0.1 ^a b f^	5.6 ± 0.1 ^a b f^	5.5 ± 0.1 ^a b f^
Erythrocytes (MCV) (fl)	SP	85.1 ± 0.8	85.9 ± 0.8	85.2 ± 0.9	85.6 ± 1.0
	SE	87.4 ± 0.7 ^b f^	89.6 ± 0.9 ^a b d e^	88.7 ± 0.7 ^a b e f g^	87.8 ± 0.9 ^b g^
	TP	85.5 ± 0.7	87.9 ± 0.8 ^a b d e^	86.7 ± 0.6 ^a^	86.9 ± 0.7 ^a^
	TE	85.6 ± 0.7	88.6 ± 0.7 ^a b d e^	86.5 ± 0.6	85.6 ± 0.4
Erythrocyt (RDW)	SP	0.133 ± 0.002	0.132 ± 0.001 ^d^	0.128 ± 0.002 ^a^	0.131 ± 0.001
	SE	0.129 ± 0.002	0.141 ± 0.002 ^a b d e f^	0.131 ± 0.002 ^f^	0.129 ± 0.003 ^f^
	TP	0.124 ± 0.002 ^b^	0.129 ± 0.002 ^a d e^	0.124 ± 0.002	0.123 ± 0.002 ^b^
	TE	0.131 ± 0.002 ^f^	0.144 ± 0.003 ^a b d e f^	0.135 ± 0.003 ^b f^	0.133 ± 0.003 ^f^
Reticulocytes (10^9/l)	SP	52.7 ± 3.4	58.2 ± 5.0	41.3 ± 3.1 ^a c^	55.0 ± 4.2
	SE	55.1 ± 5.1	116.9 ± 9.9 ^a b e f^	58.6 ± 5.5 ^b c^	34.6 ± 6.5 ^a b f^
	TP	51.7 ± 4.6	57.6 ± 4.9	45.3 ± 3.3 ^c^	56.7 ± 4.3
	TE	49.4 ± 4.0	115.1 ± 10.1 ^a b e f^	63.0 ± 2.6 ^a b c f^	28.3 ± 4.4 ^a b f^
Reticulocytes (%)	SP	1.1 ± 0.1	1.1 ± 0.1	0.8 ± 0.1 ^a c^	1.1 ± 0.1
	SE	1.1 ± 0.1	2.2 ± 0.2 ^a b e f^	1.1 ± 0.1 ^b c^	0.7 ± 0.1 ^a b f^
	TP	1.1 ± 0.1	1.2 ± 0.1 ^d^	1.0 ± 0.1	1.1 ± 0.1
	TE	1.0 ± 0.1	2.1 ± 0.2 ^a b e f^	1.1 ± 0.0 ^b c^	0.5 ± 0.1 ^a b f^
Iron (μmol/l)	SP	20.1 (15.5–24.6)	20.8 (16.0–25.5)	15.4 (11.9–18.9) c	21.4 (16.5–26.2)
	SE	16.8 (12.9–20.6)	15.9 (12.3–19.5) ^e f^	14.3 (11.0–17.5) ^e f^	21.6 (16.7–26.5)
	TP	21.6 (16.9–26.2)	25.5 (20.0–30.9)	20.0 (15.7–24.3)	25.0 (19.6–30.4)
	TE	15.1 (11.5–18.7) ^f^	20.4 (15.5–25.3)	13.9 (10.5–17.2) ^c f^	23.0 (17.4–28.5) ^a^
Iron saturation (%)	SP	37.3 ± 6.3	34.2 ± 5.5	26.1 ± 1.8	32.7 ± 4.2
	SE	28.4 ± 2.9	25.8 ± 4.5	25.0 ± 4.3	34.2 ± 2.7
	TP	35.9 ± 2.2	38.1 ± 2.5	35.7 ± 4.3	36.2 ± 2.2
	TE	25.6 ± 3.3	31.5 ± 4.6	29.0 ± 8.1	37.6 ± 2.7
Transferrin (μmol/l)	SP	31.3 ± 1.8	34.3 ± 2.0 ^a^	30.9 ± 2.2 ^c^	35.6 ± 2.0 ^a g^
	SE	30.6 ± 1.2	34.7 ± 1.6 ^a d e^	31.1 ± 1.3	32.3 ± 1.7 ^a^
	TP	30.7 ± 1.3	34.3 ± 1.3 ^a^	30.1 ± 0.7 ^c^	35.0 ± 1.1 ^a g^
	TE	31.3 ± 1.5	35.6 ± 1.4 ^a d e^	30.4 ± 1.5	30.9 ± 1.0 ^b f^
Ferritin (μg/l)	SP	115.8 ± 20.1	107.0 ± 17.8	113.9 ± 20.3	90.2 ± 14.9
	SE	135.6 ± 41.2	57.2 ± 16.6 ^a e f^	80.3 ± 24.4 ^a^	162.4 ± 48.0 ^a b f^
	TP	159.4 ± 24.4	121.1 ± 20.7 ^a b e^	113.5 ± 20.4 ^a^	88.9 ± 16.3 ^a^
	TE	112.5 ± 12.4	44.1 ± 5.2 ^a b e f^	52.1 ± 10.5 ^a e f^	141. 4 ± 7.6 ^a^
Haptoglobin (g/l)	SP	0.95 (0.70–1.20)	0.93 (0.68–1.18)	0.99 (0.73–1.26)	0.80 (0.58–1.01)
	SE	0.76 (0.57–0.95)	0.80 (0.59–1.01)	0.73 (0.54–0.91)	0.78 (0.58–0.98)
	TP	0.85 (0.62–1.08)	0.77 (0.57–0.98)	0.83 (0.61–1.05)	0.94 (0.69–1.19)
	TE	0.93 (0.67–1.20)	0.62 (0.45–0.80)	0.65 (0.47–0.84)	0.78 (0.56–1.00)

Data are expressed as mean ± SE or median (CI95%). Level of significance was p<0.05. SP; Sedentary-placebo, SE; Sedentary-ESA, TP; Training-placebo, and TE; Training-ESA. Different from baseline within group (a), SP within same time-point (b), from 3 and 13 weeks within same group (c), from 10 weeks within group (d), from 13 weeks within group (e), from TP within same time-point (f), and from TE within same time-point (g). MCHC: mean corpuscular hemoglobin concentration, MCV: mean corpuscular volume, RDW: red blood cell distribution width, EVF: erythrocyte volume fraction, MCH: mean corpuscular hemoglobin.

### Serum proteome pattern

**Fig 2:**
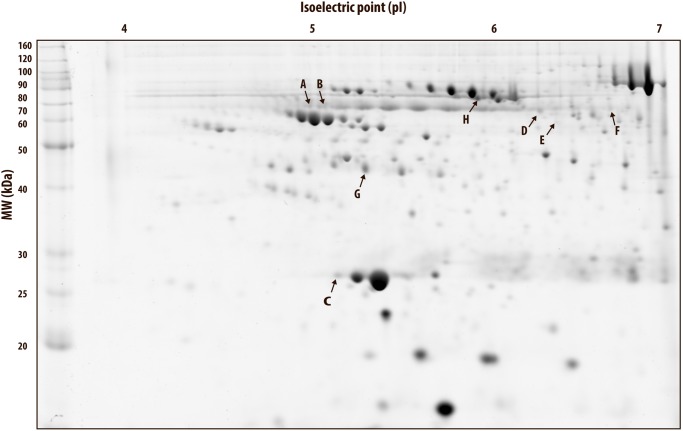
Representative 2DE gel with significant spots. Representative 2-dimensional electrophoresis (2DE) gel of human serum. Protein spots that changed significantly (p<0.05) are marked.

The serum proteome pattern in all gels was homogeneous and consistent in each subject throughout the study period. A representative 2DE gel is presented in Fig. 2. A total of 125 spots were detected in the serum 2DE gels of which 80 were matched in all gels. Only spots that were matched in all gels were used for further statistical analysis. For implementation of a spot in the blood passport, it is important that the intensity is high enough for detection in all subjects, in order to observe possible changes.

### Serum proteome changes after Darbepoietin-α treatment

**Fig 3:**
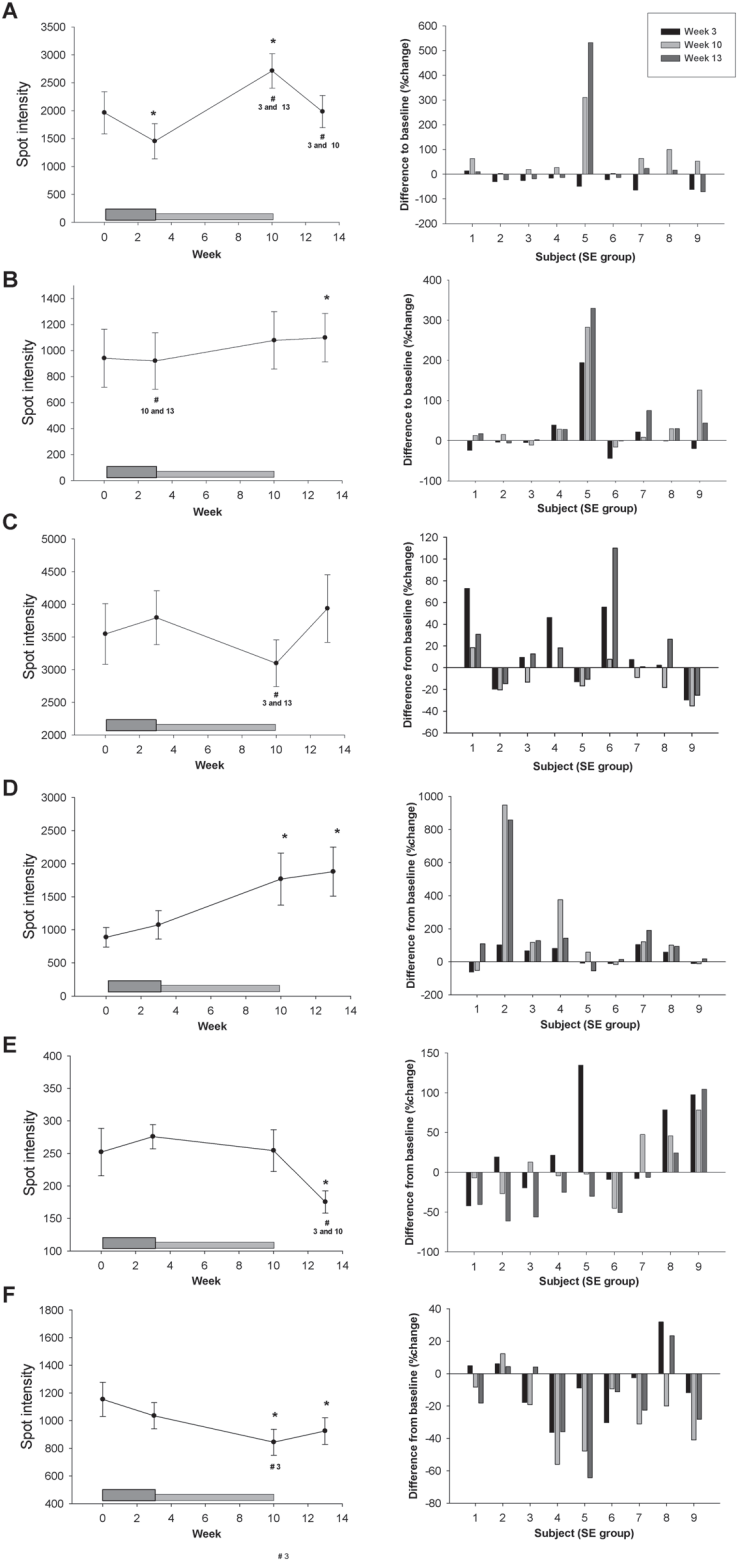
Spots affected by Darbepoietin-α treatment alone. A total of six spots changed significantly in relation to Darbepoietin-α treatment alone, two graphs for each of the spots A-F are shown. Left panel show the average change in spot intensity for the sedentary-ESA group (n = 9). The dark grey box at the bottom indicates the boosting period (first 3 weeks) and the light grey box the maintenance period (weeks 4–10), subjects were not treated during weeks 11–13 (wash-out phase). Data are expressed as mean ± SE, level of significance are p<0.05, * indicates difference from baseline (week 0) and # difference from the week number given below. Right panel shows individual changes (%) from baseline in all subjects. Black bars; week 3, light grey; week 10, dark grey; week 13.

In order to isolate the effect of Darbepoietin-α treatment and to be able to compare with our previous study with 16 days of rHuEpo treatment, we first analyzed the changes in spot intensities during the intervention period in the sedentary-ESA group alone. Six spots (A-F) exhibited significantly changed levels during the study period in relation to Darbepoietin-α treatment alone [One-way ANOVA, overall time effect, A; p<0.001 (~70 kDa, isoelectric point (pI) ~5), B; p = 0.038 (~70 kDa, ~5.1 pI), C; p = 0.039 (~27 kDa, ~5.2 pI), D; p = 0.032 (~65 kDa, ~6.3 pI), E; p = 0.036 (~58 kDa, ~6.4 pI), and F; p = 0.013 (~66 kDa, ~6.7 pI), Figs. 2–3]. In order to implement these protein isoforms in the biological passport in the future, it is important that the same changes are observed in all subjects. Thus, the individual percentage changes from baseline are shown in Fig. 3. Only spot A, D, and F showed approximately the same pattern in all nine subjects during the treatment period (Fig. 3), however, the variation is probably still too large for these to be good biomarkers.

**Fig 4:**
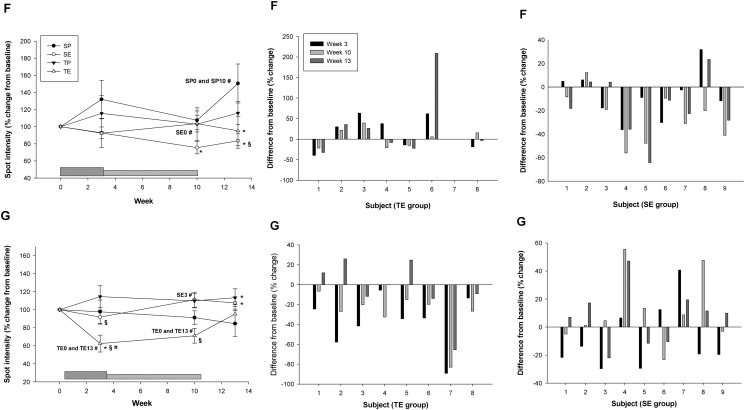
Spots with an overall Darbepoietin-α effect. For the spots G and F that exhibited a significant Darbepoietin-α effect are shown average change in spot intensity. SP (sedentary-placebo), SE (sedentary-ESA), TP (training-placebo), and TE (training-ESA). The dark grey box at the bottom indicates the boosting period (first 3 weeks) and the light grey box the maintenance period (weeks 4–10), subjects were not treated during weeks 11–13 (wash-out phase). Individual changes (%) from baseline in all subjects in the TE and SE group are also shown. Data are expressed as mean ± SE, level of significance are p<0.05. * significant from SP within same time-point, # significant from different time-point within group (time point are given), § significant from TP within same time-point, ¤ significant from SE within same time-point.

Secondly, the changes in all four groups were considered in order to evaluate the interaction between Darbepoietin-α treatment and aerobic training. With this approach only two spots displayed a significant change attributable to Darbepoietin-α alone (Three-way ANOVA): spot F (ESA effect, p = 0.046) and spot G (~45 kDa, ~5.5 pI) (ESA*Time effect, p = 0.006) (Fig. 4). During the intervention period all subjects in the combined training-ESA group showed a significant decrease in spot intensity for spot G. The pattern is not as clear for spot F (Fig. 4). Thus, spot G exhibited the most robust changes.

### Serum proteome changes after aerobic training

**Fig 5:**
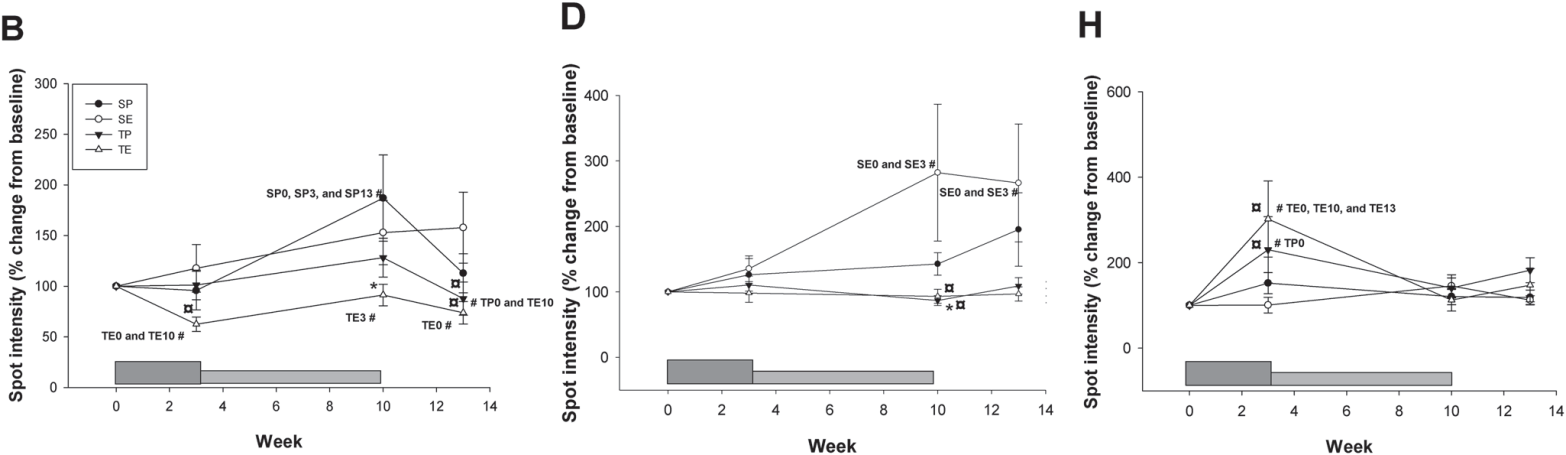
Spots with overall training effect. Average spot intensity for spots B, D, and H, which showed an overall training effect. SP (sedentary-placebo), SE (sedentary-ESA), TP (training-placebo), and TE (training-ESA). The dark grey box at the bottom indicates the boosting period (first 3 weeks) and the light grey box the maintenance period (weeks 4–10), subjects were not treated during weeks 11–13 (wash-out phase). Data are expressed as mean ± SE, level of significance are p<0.05. * significant from SP within same time-point, # significant from different time-point within group (time point are given), ¤ significant from SE within same time-point.

It is important to consider the isolated effects of training on potential biomarkers for Darbepoietin-α abuse and to identify markers that are affected only by Darbepoietin-α. When analyzing the training-placebo group alone, none of the identified spots showed a significantly altered spot intensity during the intervention period. When including all four groups, a significant training effect was found for three spots: B (training effect, p = 0.017 and time*training effect, p = 0.019), D (time*training effect, p = 0.036), and H (~72 kDa, ~5.9–6 pI) (training effect, p = 0.006, and time*training effect, p = 0.001) (Fig. 5). Thus, these three spots (B, D, and H) are not optimal biomarkers for Darbepoietin-α abuse.

### Identification of protein identities

LC-MS/MS was performed in order to determine the protein identities of significantly altered spots (Table 3). The depletion kit used only removes approximately 85% of IgG and albumin; thus, some IgG and albumin may still be present in the samples. It is presumed that the amounts of albumin and/or IgG are low and do not contribute significantly to the observed spot volume changes.

Darbepoietin-α treatment alone seemed to affect spots containing; kininogen-1 (spot A), anti-thrombin-III (spot A), alpha-2-antiplasmin (spot A+B), angiotensinogen (spot A), alpha-1B-glycoprotein (spot B), apolipoprotein A-I (spot C), beta-2-glycoprotein 1 (spot D), and serotransferrin (spot E). When looking at all 4 intervention groups Darbepoietin-α showed a significant effect on two spots containing serotransferrin (spot F) and haptoglobin/haptoglobin related protein (spot G), respectively. Training on the other hand affected spots containing; alpha-2-antiplasmin (spot B), alpha-1B-glycoprotein (spot B), beta-2-glycoprotein 1 (spot D), and hemopexin (spot H) (Table 3).

**Table 3:** Protein identities (LC-MS/MS analysis).

**Spot #**	**Protein**	**Peptides**	**Mascot Score**	**Mr (kDa)**	**Sequence coverage (%)**	**Identification**
A	Kininogen-1	ENFLFLTPDCK	59	73	7.0	KNG1_HUMAN
		YNSQNQSNNQFVLYR	50			
		DIPTNSPELEETLTHTITK	92			
	Antithrombin-III	EVPLNTIIFMGR	57	53	6.0	ANT3_HUMAN
		EQLQDMGLVDLFSPEK	75			
	Alpha-2-antiplasmin	LCQDLGPGAFR	40	55	2.2	A2AP_HUMAN
	Angiotensinogen	ALQDQLVLVAAK	51	53	2.5	ANGT_HUMAN
	*Ig heavy chain V-III region VH26 or CAM*	NTLYLQMNSLR	45	13	10.3	HV303_HUMAN or HV307_HUMAN
B	Alpha-2-antiplasmin	LCQDLGPGAFR	57	55	5.5	A2AP_HUMAN
		LVPPMEEDYPQFGSPK	91			
	Alpha-1B-glycoprotein	HQFLLTGDTQGR	55	55	2.4	A1BG_HUMAN
	*Serum albumin*	LVNEVTEFAK	50	70	1.6	ALBU_HUMAN
C	Apolipoprotein A-I	VQPYLDDFQK	52	31	13.9	APOA1_PONAB
		DYVSQFEGSALGK	85			
		LLDNWDSMTSTFSK	39			
	Apolipoprotein A-I	VQPYLDDFQK	52	31	8.6	APOA1_HUMAN
		DYVSQFEGSALGK	85			
D	Beta-2-glycoprotein 1	KATVVYQGER	43	40	2.9	APOH_HUMAN
	*Ig alpha-1 chain C region*	TPLTATLSK	65	39	9.6	IGHA1_HUMAN
		WLQGSQELPR	56			
		DASGVTFTWTPSSGK	42			
E	Serotransferrin	DSGFQMNQLR	66	79	9.3	TRFE_HUMAN
		SVIPSDGPSVACVK	44			
		MYLGYEYVTAIR	81			
		EDPQTFYYAVAVVK	76			
		IECVSAETTEDCIAK	56			
	*Ig gamma-2 or gamma-4 chain C region*	STSESTAALGCLVK	85	37	4.3	IGHG2_HUMAN or IGHG4_HUMAN
F	Serotransferrin	HSTIFENLANK	56	79	12.5	TRFE_HUMAN
		MYLGYEYVTAIR	63			
		CSTSSLLEACTFR	45			
		DQYELLCLDNTR	42			
		KPVEEYANCHLAR	69			
		EGTCPEAPTDECKPVK	66			
	*Ig alpha-1 chain C region*	QEPSQGTTTFAVTSILR	122	39	4.8	IGHA1_HUMAN
G	Haptoglobin	SCAVAEYGVYVKNLNEKDYELLCLDGTR	65	46	15.3	HPT_HUMAN
		YVMLPVADQDQCIR	90			
		VMPICLPSKDYAEVGR	91			
		SPVGVQPILNEHTFCAGMSK	137			
	Haptoglobin related protein	SCAVAEYGVYVK	65	40	9.2	HPTR_HUMAN
		SPVGVQPILNEHTFCVGMSK	41			
H	Hemopexin	GECQAEGVLFFQGDR	79	52	15.2	HEMO_HUMAN
		LLQDEFPGIPSPLDAAVECHR	41			
		EVGTPHGIILDSVDAAFICPGSSR	72			
	*Serum albumin*	LVNEVTEFAK	66	71	14.0	ALBU_HUMAN
		FKDLGEENFK	76			
		AVMDDFAAFVEK	70			
		AAFTECCQAADK	88			
		CCAAADPHECYAK	60			
		KVPQVSTPTLVEVSR	48			
		QNCELFEQLGEYK	125			
	*Ig alpha-1 chain C region*	QEPSQGTTTFAVTSILR	62	39	4.8	IGHA1_HUMAN

The protein(s) found in each spot are shown. Proteins that have been tried depleted from the samples before the 2DE analysis are shown in italic. Significant identified peptides are shown together with their Mascot Score, a score >38–39 (protein dependent) is considered significant (p<0.05). The calculated molecular mass, based on the amino acid sequence, and the sequence coverage, the percentage of the total protein that the significant peptides cover, are shown. M = oxidized M. Mascot: http://www.matrixscience.com.

### Serum haptoglobin

**Fig 6:**
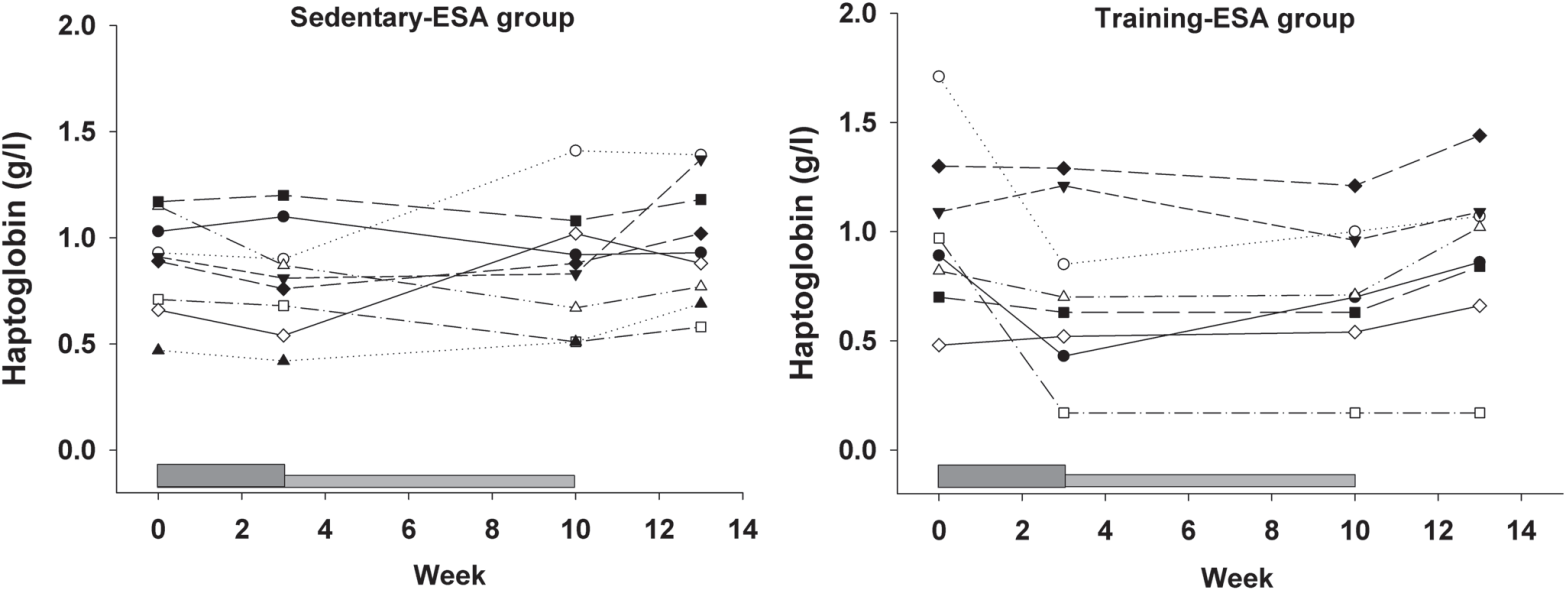
Total serum haptoglobin levels. The individual total serum haptoglobin levels in the sedentary-ESA and training-ESA groups, respectively, throughout the study period. The dark grey box at the bottom indicates the boosting period (first 3 weeks) and the light grey box the maintenance period (weeks 4–10), subjects were not treated during weeks 11–13 (wash-out phase).

Darbepoietin-α treatment tended to decrease total serum haptoglobin levels (Time*ESA, p = 0.053) (table 2). The individual changes in each subject in the two ESA groups are shown in Fig. 6.

## Discussion

In the current study, we investigated the changes in serum proteomics in healthy untrained men after Darbepoietin-α treatment and/or aerobic training. We observed that six protein spots changed significantly during the combined intervention and washout period in response to Darbepoietin-α treatment alone. When comparing all four experimental groups, two protein spots, containing isoforms of serotransferrin and haptoglobin/haptoglobin related protein, showed a significant response to Darbepoietin-α treatment. The spot containing an isoform of haptoglobin/haptoglobin related protein showed a significantly lower intensity in all subjects in the training-ESA group during the Darbepoietin-α treatment period, and an increase in intensity during the washout period. However, the total serum haptoglobin/haptoglobin related protein levels did not change significantly, emphasizing the importance of monitoring the individual isoforms of a protein. Thus, this isoform of haptoglobin/haptoglobin related protein could be a potential new anti-doping marker. The isoform of serotransferrin on the other hand did not show as clear a pattern as the haptoglobin/haptoglobin related protein isoform.

### Hematological measures and aerobic capacity

Both Darbepoietin-α treatment and aerobic training, and both in combination, significantly increased the maximal oxygen uptake, as expected. Darbepoietin-α treatment for 10 weeks resulted in an initial increase in reticulocyte levels followed by an increase in hemoglobin and hematocrit levels, the latter two being stable throughout the last part of the treatment period. Even though the subjects were supplemented with iron, serum iron levels decreased significantly throughout the study. However, the levels were still within the normal range and, therefore, should not have affected the erythropoiesis.

### Proteomic changes

The proteomic data in the current study showed that several protein spots were altered in relation to Darbepoietin-α treatment alone. These spots contained kininogen-1, antithrombin-III, alpha-2-antiplasmin, angiotensinogen, alpha-1B-glycoprotein, apolipoprotein A-I, beta-2-glycoprotein 1, and serotransferrin. When taking all four experimental groups into consideration, the intensity of only two spots, containing mainly haptoglobin/haptoglobin related protein and serotransferrin were significantly altered after Darbepoietin-α treatment. Compared to our previous study on proteomic changes in response to rHuEpo treatment [9], we confirmed that spots containing haptoglobin and serotransferrin respond to Darbepoietin-α /rHuEpo treatment.

In the current study, spot G containing haptoglobin/haptoglobin related protein seemed to decrease in intensity in response to treatment in the same manner in all subjects in the training-ESA group. However, the same clear pattern was not found in the groups that received Darbepoietin-α treatment or endurance training alone, thus, in the current study, it seems that both training and Darbepoietin-α treatment are needed to induce changes in spot G. In support of this spot being Epo responsive, we have previously shown a decrease in haptoglobin isoforms in the ~45kDa train [9], corresponding to spot G in the current study. Together, our results indicate that haptoglobin does change with Darbepoietin-α/rHuEpo treatment and that this change is not affected by endurance training alone. Furthermore, there was a tendency to a Darbepoietin-α-induced decrease in total serum haptoglobin levels. This is in accordance with the significant decrease in total serum haptoglobin, we have previously shown after 16 days of rHuEpo treatment [9].

Haptoglobin is a serum protein that binds free hemoglobin and thereby prevents hemoglobin-related oxidative damage. The haptoglobin-hemoglobin complex is removed from the circulation by endocytosis into monocytes and tissue macrophages and finally degraded in the liver and spleen; in that way iron loss through the kidneys is prevented [15]. Free haptoglobin has a half-life of approximately 5 days and serum levels are relatively constant, however, after binding to hemoglobin the half-life of the complex is shortened to minutes. Haptoglobin is not recycled after endocytosis of the hemoglobin-haptoglobin complex, leading to rapid depletion of haptoglobin in individuals with increased free hemoglobin. An association with low haptoglobin and acute hemolysis has been demonstrated in a large cohort of patients with various hemolytic diseases [16]. In addition, physiological adaptations in order to down-regulate the red blood cell mass if it is excessive has been described. In humans, neocytolysis is a process that are initiated when red blood cell mass is too high and endogenous Epo production is suppressed, in which there is an accelerated hemolysis of the youngest circulating red blood cells [17, 18]. In response to return from space flights or descent from high altitude a reduction of 10–15% in red blood cell mass have been reported within a week [17]. Furthermore, in mice overexpressing the human Epo gene with a hematocrit up to 90%, the life span of the erythrocytes was markedly reduced to one third, counteracting the fatal effects of excessive erythropoiesis. Two mechanisms were proposed; 1) the erythrocytes aged faster than normal erythrocytes resulting in enhanced phagocytosis, 2) increased number and phagocytic activity of the macrophages [19]. It could therefore be hypothesized that an increased turnover of red blood cells, due to the higher hematocrit, results in the formation of more haptoglobin-hemoglobin complexes, which will be degraded and result in a decrease in free serum haptoglobin. Perhaps haptoglobin/haptoglobin related protein (spot G) represents an isoform of particular importance for this activity. Further research is needed to confirm this.

Serotransferrin is the major transporter of iron from its storage sites to the bone marrow. Spot F containing serotransferrin also showed the same pattern in most subjects, although not as consistent as spot G. Furthermore, the intensity of spot F decreased significantly after Darbepoietin-α treatment also when comparing the changes in all 4 groups. Isoforms of serotransferrin were also found to be significantly decreased after 16 days of rHuEpo in our previous study [9]. Due to different separation techniques in the two studies, it is difficult to assess if the location of the isoforms on the 2DE gel are the exact same between the two studies. However, total serum serotransferrin levels actually increased after the boosting period, exhibiting the opposite pattern of spot F. This opposite changes in total serum levels of serotransferrin versus the individual isoforms in response to rHuEpo was also found in our previous study [9]. We do currently not have an explanation for this, but the results highlight the important fact that all isoforms of a protein do not respond in the same manner to a particular intervention. Different isoforms of a protein usually display mass and/or charge shifts generated primarily by posttranslational modifications (PTM), such as protein cleavage and side-chain residue modifications (e.g. phosphorylation, sulfonation, oxidation, and glycosylation among others) [8]. These modifications may induce change in the activity of the modified protein.

Several of the significantly changed spots identified here contained more than one protein (spot A, B, D, E, F, G, H). In such cases it is difficult to state which of the proteins are responsible for the altered intensity. This is even more complicated when the spot intensity is low and only a few peptides are identified for each protein. In cases where numerous peptides are identified in the same sample, it is likely that the protein with the most identifications are also present in the highest concentration, and it is more likely that this protein is responsible for the observed changes in intensity.

Six spots showed significantly altered spot intensity when evaluating the sedentary-ESA group alone; one of these was spot F, discussed above. Even though the individual changes in spot A and B were quite similar in all subjects, the identification of numerous proteins in these spots makes interpretation difficult. By contrast, spot C, D, and E contained only one protein. Although the intensity of spot C and E significantly changed over time, a considerable inter-individual variation was evident. The inter-individual variation was low for spot D, but this spot was also affected by aerobic training (see below).

In addition to changes induced by Darbepoietin-α, we also identified 3 spots (B, D, and H) that responded significantly to aerobic training. It is relevant to consider proteins that change in response to aerobic training, as these proteins are not suitable as anti-doping markers. Two of these three spots (B and D) also changed in relation to Darbepoietin-α treatment alone; spot H, corresponding to hemopexin has previously been shown to change with rHuEpo treatment [9].

### Window of detection

It is also relevant to consider the window of detection when evaluating possible new biomarkers. A single dose of rHuEpo results in a 3-day window of detection for the drug in urine and the window is approximately 7 days for Darbepoetin-α, which was used in the current study [20, 21]. Indirect testing of Epo abuse by the Athlete Biological Passport significantly increases the window of detection. In this approach, hematological values are monitored over time and suspicious patterns are used to identify doping [4]. In the current study, the last Darbepoietin-α injection was given 1 week prior to performing the last measurements after the treatment period. Darbepoietin-α is detectable in serum for up to 7 days and thus, also posses physiological effects during this time period. The washout period in the current study, thus, is defined as the period with no circulating Darbepoietin-α, in some other studies it is defined as the period after the last injection. Regarding spot G, the intensity of this spot increased during the washout period and was not significantly different from baseline 3 weeks after the treatment period. Thus, the window of detection might only be 1–2 weeks after Darbepoietin-α is cleared from the blood; this is however still 1–2 weeks longer than the direct test. Further research with more frequent blood samples during the washout period is needed to establish the precise timeframe. However, a pattern during the treatment and washout period was observed for all subjects. Spot G significantly decreased in response to Darbepoietin-α treatment and peaked at either week 3 or 10; in six out of eight subjects a small increase was observed when shifting from high-dose (first 3 weeks) to low-dose treatment (week 4–10). In all subjects, this was followed by an additional increase during the washout period (week 13 > week 10). Therefore, if monitored over time, as in the hematological passport, it might be possible to reveal exposure to Darbepoietin-α /rHuEpo at least in the ON-phase.

### Limitations

It has to be established if the biomarkers identified in untrained subjects in the current study respond to the same extent in elite athletes. Furthermore, the effect of micro-dosing on these biomarkers also needs to be investigated, since many athletes to day use this strategy to circumvent being tested positive. In the current study Darbepoietin-α was administrated, it has to be established if other types of rHuEpo affect these biomarkers in the same manner. In support hereof, we have previously shown that epoietin-β induce some of the same alterations in serum proteome [9].

In the current study, the participants were given iron supplementation. It is unknown if this has affected our results, but all 4 groups were treated with iron supplementation, thus, the differences in response between our control group and the 3 experimental groups are not due to iron. Furthermore, all athletes abusing Epo also take iron supplementation in order to secure maximum effect of the Epo treatment.

### Perspectives

Spot G corresponding to an isoform of haptoglobin/haptoglobin related protein seems to be the most promising anti-doping marker identified in the current study. It is interesting from a physiological point of view to understand what distinguishes this particular isoform of haptoglobin/haptoglobin related protein from the other isoforms with approximately the same molecular weight. In addition, information of the chemical nature of the corresponding post-translational modifications is also greatly needed. This information is needed in order to develop antibodies specific for this particular isoform of haptoglobin, which would make its detection both easier and faster.

Before implementing haptoglobin as a biomarker, the effects of hypoxia/altitude also have to be established. Hypoxia is a strong stimulus to increase endogenous Epo production, which is why athletes frequently live and train at high altitude in order to improve their aerobic capacity. Previous studies in humans [22] and in a human hepatoma cell line [23] exposed to hypoxia found that haptoglobin expression was up-regulated; opposite the tendency to lower serum haptoglobin levels found in relation to Darbepoietin-α/rHuEpo treatment in our studies. The reason for these discrepancies is currently unknown, further emphasizing the importance of evaluating the effect of altitude/hypoxia on these serum biomarkers. Furthermore, the effect of an acute strenuous bout of exercise on serum proteomics also needs to be evaluated.

In conclusion, it is possible by a proteomic approach to identify protein isoforms in serum that change significantly in response to Darbepoietin-α exposure without interaction by time or physical activity. Of particular interest, a haptoglobin/haptoglobin related protein isoform was identified that responded robustly to Darbepoietin-α administration as a replication of observations made in a previous study [9]. The chemical nature of this particular isoform merits detailed characterization in future studies.

## Supporting Information

S1 CONSORT Checklist (DOC)

S1 Protocol Protocol approved by the Local Human Ethical Committee of Central Denmark Region in Danish. (DOC)

S2 Protocol Protocol approved by the Local Human Ethical Committee of Central Denmark Region in English. (DOCX)
