# Supplementary material for: Serum Proteomic Changes after Randomized Prolonged Erythropoietin Treatment and/or Endurance Training: Detection of Novel Biomarkers
Source: PLoS One. 2015 Feb 13;10(2):e0117119. doi: 10.1371/journal.pone.0117119 (PMC4332672; doi:10.1371/journal.pone.0117119)
Supplement: S1 Protocol — (DOC) [file pone.0117119.s002.doc]

Sagsnr. M-20110035

**Identifikation af nye serum markører til detektion af misbrug med erythropoietin**

Britt Christensen, Birgitte Nellemann og Jens Otto Lunde Jørgensen

Medicinsk Endokrinologisk Afdeling MEA og Medicinsk Forskningslaboratorium, Århus Universitetshospital, Århus Sygehus, Nørrebrogade 44, DK-8000 Århus C

Tlf.: +45 89 49 20 35

**Samarbejdspartnere**

- Medicinsk Forskningslaboratorium

Århus Sygehus
Nørrebrogade 44
8000 Århus C

- Medicinsk Endokrinologisk Afdeling

Århus Sygehus
Nørrebrogade 44
8000 Århus C

- Institut for Idræt

Århus Universitet

Dalgas Avenue 4

8000 Århus C

**Tidsplan**

Forventet start: Juni 2011

Forventet afsluttet: August 2012

Sted

Medicinsk Forskningslaboratorium, Århus Sygehus.

Institut for Idræt, Århus Universitet.

**Baggrund**

Erytropoietin (EPO) er et ca. 34 kDa glykoprotein, der syntetiseres i nyrerne som respons på lav ilttension. EPO stimulerer nydannelsen af erytrocytter i knoglemarven, samtidig med at plasma volumen nedsættes (Lundb*y et a*l., 2007). Herved øges ilt bindingskapaciteten i blodet. Rekombinant humant EPO (rHuEPO) kom første gang på markedet i 1989, og benyttes i dag som lægemiddel mod uræmisk anæmi og cancer induceret anæmi.

Idet EPO stimulerer produktionen af erytrocytter, og injektion med rHuEPO over længere tid øger den submaksimale kapacitet med mere end 50% (Thomse*n et a*l., 2007), bliver det til stadighed misbrugt af atleter i udholdenheds sportsgrene (Barros*o et a*l., 2008;Pascua*l et a*l., 2004;Catli*n et a*l., 2008). Det på trods af at rHuEPO kom på anti-doping listen allerede i 1990. Det er derfor yderst vigtigt at finde en sensitiv og robust metode til at detektere dette misbrug. Ikke kun for at sikre en fair konkurrence, men også for at mindske misbruget og dermed de bivirkninger dette medfølger såsom øget risiko for kardiovaskulær sygdom.

Den eksisterende metode til at detektere rHuEPO, godkendt af WADA, er baseret på forskelle i glykosylerings grad og mønster, mellem rHuEPO og det endogene EPO (Catli*n et a*l., 2008;Pascua*l et a*l., 2004). Denne metode er dog ufølsom og relativ dyr (Pascua*l et a*l., 2004;Gor*e et a*l., 2003). Om denne metode vil være effektiv mod nye generationer af EPO derivater, der er produceret i humane celle linjer og dermed udtrykker den samme glykosylerings grad og mønster som rHuEPO, er stadig ukendt (Gor*e et a*l., 2003). Herudover er gen-doping også en fremtidig doping strategi, man bliver nødt til at forholde sig til. Stabil transfektion af EPO genet i væv er allerede vist i dyre modeller (Hojma*n et a*l., 2007). Gen-doping blev således i 2003 tilføjet WADA og IOCs liste over ”Prohibited substances and methods in sport” (Pascua*l et a*l., 2004). Dette understreger vigtigheden af at udvikle nye og mere sensitive EPO analyser. Fremtidige anti-doping tests bør derfor ikke kun fokusere på at detektere rHuEPO og derivater heraf, men også på biologiske markører der ændres ved EPO eksponering. Sådanne markører har været undersøgt. Man har bl.a. kigget på total hæmoglobin masse og retikolocyt procentdelen (OFF_hr-score_) samt det såkaldte blod-pas (Gor*e et a*l., 2003). Problemet med OFF_hr-score_ er dog, at cut-off værdierne er baseret på det gennemsnitlige populations niveau, og pga. store forskelle individer imellem bliver cut-off værdierne (øvre og nedre grænse) meget brede for at undgå falsk-positive resultater. Dette fører ultimativt til et lavt detektionsniveau (Lundby & Robach, 2009;Born*o et a*l., 2010). Også blod-passet, hvor man følger atleters blodværdier over tid, har vist sig ikke at garantere en doping fri sport (Born*o et a*l., 2010).

Proteomics har vist sig at være en god metode til at karakterisere alle proteinerne udtrykt i et biologisk system; såsom blodet. Vi har allerede i et tidligere studie vist, at 16 dages behandling med rHuEPO medførte signifikante ændringer i en række serum proteiner (Christense*n et a*l., 2010). Hovedformålet med dette projekt er at undersøge effekten af længerevarende rHuEPO behandling på ændringer i serum proteomet hos raske unge mænd. Vi ønsker ydermere at undersøge effekten af rHuEPO og fysisk aktivitet på disse markører, idet det er vigtigt at belyse effekten af fysisk aktivitet i sig selv, hvis disse markører skal bruges som anti-doping markører.

EPO receptorer findes i mange forskellige typer væv ud over knoglemarven, bl.a. neuroner, astrocytter, microglia, cancerceller, lydingceller, gastriske mucosale celler og ikke mindst muskel væv. Effekten af EPO i disse væv mangler dog stadig at blive fuldt belyst.

Vi ønsker sekundært i dette studie at kigge på, hvilke effekter EPO har på fedt- og muskelvæv samt eventuelle effekter på substratmetabolisme og insulinfølsomhed. Vi har i et tidligere studie af rHuEPOs akutte effekter vist, at EPO muligvis har en effekt på fedtomsætningen (upublicerede data). Studiet viste også, at EPO receptorer findes i muskel væv, og at længerevarende behandling med rHuEPO medfører ændringer i muskelproteomet. Dette tyder på, at EPO muligvis inducerer et skift i muskelfiber type fra en hurtig glykolytisk fænotype til en langsommere fænotype (data submittet).

**Formål**

At identificerer nye serum biomarkører for at kunne detektere misbrug af EPO. Sekundært at undersøge effekten af 10 ugers EPO behandling samt træning på en lang række andre parametre, såsom omsætningen af fedt, kulhydrat og protein, muskeltype ændringer, aktivering af satellitceller i musklerne mm.

**Design**

- Forsøget er et enkelt blindet og randomiseret studie.
- I alt inkluderes 40 forsøgspersoner som randomiseres til en af følgende grupper (n=10);
  - Placebo behandling
  - Placebo behandling + udholdenhedstræning
  - rHuEpo behandling
  - rHuEpo behandling + udholdenhedstræning
- Forsøgspersonerne undersøges før og efter 10 ugers EPO/placebo behandling, samt efter en 3 uger udvaskningsperiode.
- EPO (Darbapoietin alpha) (≈50 IU/kg)/placebo (saltvand) indgives s.c. 2 gange om ugen de første 3 uger i dosis 2x40 μg, og en gang i ugen fra og med uge 4-10 i dosis 20 μg.
- Udholdenhedstræningen vil bestå af 1-1.5 times cykling ved 65-80% af VO_2_max 3 gange ugentligt, hvilket tidligere er vist at medføre en øgning i maksimal ilt optagelse (Midgle*y et a*l., 2006).

**
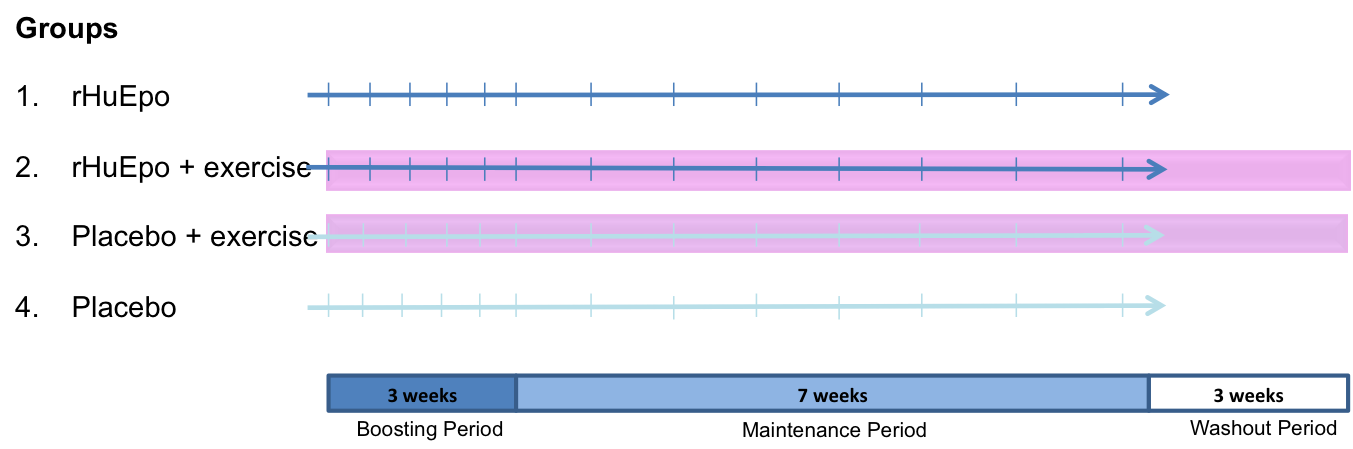
**

**Undersøgelses program:**

- Metaboliskprofil, hyperinsulinemisk euglukæmisk clamp, muskel- og fedtbiopsier, blodprøver, maksimal iltoptagelsestest, DEXA skanning

**Primære end points:** Proteomics analyser på serum

**Sekundære end points:** Substratmetabolisme, fibertype bestemmelse, aktivering af satellitceller

**Forsøgspopulationen**

I alt 40 raske unge mænd undersøges. Disse rekrutteres via www.forsøgsperson.dk og diverse opslag på uddannelsesinstitutionerne i Århus (se vedlagte opslag).

Da effekten af EPO på nogle af de ønskede parametre ikke tidligere er belyst, har det ikke været muligt at lave en power beregning. Fra lignende studier på vores forsøgslaboratorium er det beregnet, at der skal bruges ca. 10 forsøgspersoner i hver gruppe. Vi har desuden vist i et tidligere studie, at det i en gruppe på 8 forsøgspersoner var muligt at detekterer signifikante ændringer i proteomet efter 16 dages behandling med rHuEpo (Christense*n et a*l., 2010).

Inklusionskriterier:

- Skriftligt samtykke før undersøgelsesstart
- Myndige, raske mænd
- Alder > 18 år og < 35 år.
- Normalvægtige (BMI: 18-27)
- Utrænede (< 2 timers fysisk aktivitet per uge, samt kondital under 45)
- Ikke ryger

Eksklusionskriterier:

- Kronisk sygdom
- Blodtryk over 135/85
- Hæmatokrit over 45%

Deltagerne vil udgå af undersøgelsen hvis:

- De selv ønsker det
- Investigator skønner, at deltageren ikke kan leve op til forsøgsprocedurerne eller pga. sikkerhedsmæssige årsager
- Deltageren udvikler alvorlige eller intolerable bivirkninger. I så fald vil forsøget for den enkelte deltager blive stoppet og investigator vil foranledige relevant opfølgning herunder henvisning til relevant behandling, og der vurderes herefter, om forsøget som helhed skal bringes til ophør.

Deltageren må trækkes ud af forsøget på et hvilket som helst tidspunkt. Data opnået fra udgået forsøgsperson vil blive anvendt, så vidt det er muligt – det vil afhænge af, hvornår i undersøgelsen en given forsøgsperson måtte udgå. De data, det er muligt at analysere sig frem til, vil blive benyttet.

**Metoder**

**Almindelig lægelig helbredsundersøgelse:** Grundig objektiv lægelig undersøgelse der indebærer optagelse af sygdomsanamnese, måling af blodtryk, livvide, vægt, højde, EKG og rutineblodprøver. Rutineblodprøver tages forud opstart på forsøget, for at sikre at forsøgsdeltagerne er sunde og raske. Følgende vil blive målt; Hæmoglobin, erythrocytter, jern, transferrin, ferritin, reticulocytter, haptoglobin, albumin, basisk phosphatase, billirubin, LDH, plasma hæmoglobin, ALAT, kalium, natrium, creatininium, leukocytter, thrombocytter, CRP, HbA1c, cholesterol, TSH, GFR mm.

**Maksimal iltoptagelses test:** Maksimal ilt optagelses test vil blive foretaget mindst en uge inden den første metaboliske dag i forbindelse med at screeningsblodprøverne og den lægelige undersøgelse foretages. Testen vil blive fortaget igen halvvejs gennem træningsperioden og ved afslutningen af forsøget dagen efter den afsluttende metaboliske dag. Testen foretages på cykel v.h.a. indirekte kalorimetri og det tilstræbes at nå maximal iltoptagelse efter ca. 5-7 min.

**DEXA skanning:** Kropssammensætning måles ved hjælp af dual-energy-X-ray absorptiometri (DEXA) på en Hologic Discovery forud for behandlingen og ved behandlingsophør.

**Metabolisk profil:**

- **Hyperinsulinæmisk euglykæmisk clamp:** Intravenøs infusion af hurtigtvirkende insulin (Actrapid, Novo Nordisk A/S, DK) 0,6 mU/kg TBW/min og samtidig løbende intravenøs infusion af 20 % glukose opløsning, der justeres således, at p-glukose ~ 5 mM. Dette niveau af glukose sikres ved måling af blodsukker hver 5.-10. min og tilsvarende justering af glukose-infusionshastigheden. Mængden af infunderet glukose (M-værdien) er et udtryk for insulinfølsomheden.
- **Carbamidtracer:** Der gives en bolus ^13^C-carbamid (390,6 mg) efterfulgt af konstant infusion af ^13^C-carbamid (42 mg/time) i 4 timer.
- **Glukosetracer:** Der gives en bolus ^3^H_3_-glukose (20 µCi) og herefter konstant infusion (0,20 µC/min) i 6 timer.
- **Palmitattracer:** Bestemmelse af FFA omsætning foretages ved hjælp af isotopfortyndingsteknik med 2 x 1 times konstant infusion af (9,10-3H)palmitat (0,3 μCi/min svarende til i alt 18 x 2 μCi).
- **Aminosyretracere:** Der gives ^15^N-Tyrosin (0,3 mg/kg) samt ^2^H_4_-Tyrosin (0,5 mg/kg) som bolus. Herefter infunderes ^2^H_4_-Tyrosin (0,5 mg/kg/time) i 4 timer. ^15^N-Phenylalanin gives som en bolus (0,7 mg/kg) og infunderes herefter i 4 timer (0,7 mg/kg/time).

- **Undersøgelse for substratoptagelse i underarm:** Der placeres et plastkateter (venflon) retrogradt i en dyb antecubital vene til opsamling af venøst blod fra underarmsmuskulaturen. Der placeres endvidere på modsatte arm et kateter (venflon) antegradt i en vene på håndryggen samt i den antecubitale vene. Hånden lægges i varmekasse hvorved blodet arterialiseres. Kriterier for korrekt anlæggelse er oxygen saturationer på under 70 % for venøst blod og over 91 % for arterialiseret blod. Før blodprøvetagning måles bloodflowet med venøs okklusions plethysmografie. Bloodflow til hånden afbrydes med cuff (250 mmHg) lige før måling af bloodflow og 1 minut før prøvetagning fra vene. Arterielt og venøst blod bliver indsamlet simultant.
- **Indirekte kalorimetri:** Der udføres indirekte kalorimetri (Oxycon Pro, Carefusion, Germany) 30 minutter forud for og til slut i forbindelse med insulin stimulationen. Dette giver mulighed for at beregne total energi forbrug, oxidative rater for glukose og fedt, ikke-oxidativ glukose turnover og glukose output fra leveren.
- **Biopsi:** Biopsierne tages en timer efter starten af den metaboliske profil og 30 min inde i den hyperinsulinæmiske clamp. *Fedtbiopsi:* Subkutant fedt aspireres fra abdomen med en liposuction kanyle under lokalbedøvelse og noget af det straks-fryses i flydende nitrogen. Den resterende del af fedtcellerne bliver renset i kollagenase og farvet med methylenblåt til visualisering af nukleii og cellemembran. V.h.a. mikroskop og kamera tages billeder til bestemmelse af fedtcellestørrelse. *Muskelbiopsi:* En muskel biopsi tages fra vastus lateralis med en Bergström biopsinål. I lokal bedøvelse (1 % lidokain) foretages efter 10-15 min. incision gennem hud og muskelfascie ca. 15-20 cm over knæniveau. Muskelvævet aspireres og straks-fryses i flydende nitrogen eller indlejres i Tissue Tek og fryses herfter. *Analyser****:*** Western Blot, PCR, immunhistokemiske analyser, intramuskulært TG og glykogen.
- **Blodprøver:** Der tages blodprøver ca. hvert 20. minut under den metaboliske undersøgelsesdag. Samlet blodtab andrager ca. 320 ml. *Analyser:* EPO, totalt, frit og bioaktivt IGF-I, IGFBP-1, glukose, insulin, C-peptid, frie fede syrer, adiponectin, VLDL-TG, metabolitter, glukagon, kortisol, katekolaminer, albumin, phenylalanin enrichment, glukose specifik aktivitet, glycerol, laktat, ALAT, basisk fosfatase, bilirubin, protrombin index, carbamid, ghrelin, proteomics mm.
- **VLDL undersøgelser:** Disse undersøgelser vil blive fortaget før og efter de 10 ugers intervention. 1 uge før undersøgelsen udtages en 60 ml venøs blodprøve under sterile forhold. VLDL-TG vil blive isoleret ved ultracentrifugering (40000 g i 18 timer ved 10 °C) og efterfølgende mærket med 20 µCi [1-^14^C]triolein. Sterilitet vil blive sikret med dyrkning, før prøven anvendes til reinfusion. På undersøgelsesdagen vil patientens eget *ex-vivo* mærkede VLDL-TG blive infunderet (autolog infusion) indtil *steady-state* (4 timer). Herefter udtages blodprøve hvert 10. min over 30 min til bestemmelse af VLDL-TG SA og koncentration.
- **VLDL-TG oxidation:** Disse undersøgelser vil også blive foretaget før og efter de 10ugers intervension. VLDL-TG oxidationen måles ved opsamling af ekspirationsluft med hyamin-trapping til analysering for ^14^CO_2_, dette måles før starten på forsøgsdagen og 3 gange i løbet af den sidste halve time.
- **MR skanning:** Udføres til måling af den intrahepatiske og intramyocellulære lipidmængde. Leveren og en muskel (m. tibialis anterior) scannes med en kombination af MR imaging og lokaliseret proton MR spektroskopi med voxel størrelse på 3*3*3cm, hvor der benyttes 1,5T MR unit, samt breath holding teknik på 17 sek. Senest i forberedelsesrummet før indgang til skanner vil personen blive udspurgt om kontraindicerende forhold i overensstemmelse med normale adgangsprocedurer for MR-skanninger. Desuden vil deltagerne specielt blive gjort opmærksom på at tømme lommer og aflægge hårspænder og smykker. Forsøgsdeltagerne vil blive orienteret om MR-skanninger - om at det er en ufarlig teknik uden nogen strålingsrisiko. Der vil være et højt lydniveau i skannerrummet. Personerne vil blive forsynet med lyddæmpende ørepropper.

**
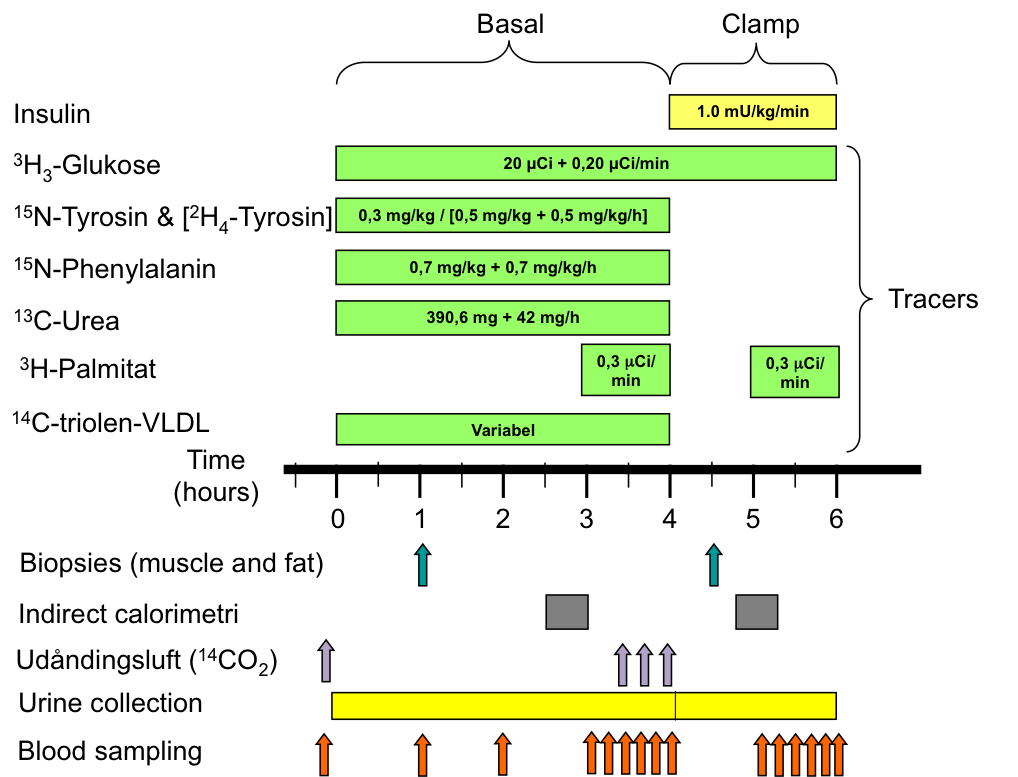
**

**Serum:** Serum vil blive taget før studiet på dag 8, 15, 21, 36, 50, 64, 71, samt på dag 78 og 92 under udvasknings perioden. Hæmatologiske niveauer vil således blive målt gennem hele studiet og behandlingen vil blive seponeret, hvis værdierne bliver unormale (hæmatokrit >55%).

Følgende vil blive målt; Hæmoglobin, erytrocytter, jern, transferrin, ferritin, retikulocytter, haptoglobin, albumin, basisk fosfatase, billirubin, LDH, plasma hæmoglobin, VLDL, FFA, Ghrelin, Cholesterol mm. Proteomics analyser vil blive foretaget på blodprøver taget før studiets start samt dag 21, 71 og 92.

**Blodtryksmålinger:** Forsøgspersonernes blodtryk vil blive monitoreret gennem hele forsøget på de dage, hvor der vil blive taget blodprøver. Dette gøres for at sikre, at behandlingen ikke inducerer forhøjet blodtryk.

Blod- og vævsprøver forventes anvendt ved analyserne og destrueres herefter. Der oprettes således ikke en biobank.

**Sikkerhedsvurdering**

Inden selve undersøgelsesdagene vil der blive foretaget en almindelig lægelig helbredsundersøgelse.

Der vil under hele forsøget være en læge og bioanalytiker tilstede, der kan foretage en almenvurdering af forsøgspersonen og foretage måling af vitale parametre (blodtryk, puls, blodsukker og bevidsthedsniveau).

Alle invasive procedurer vil blive foretaget af læge Birgitte Nellemann, og klinisk ansvarlig for projektet er professor dr.med Jens Otto Lunde Jørgensen.

Akut behandling med EPO er ikke forbundet med alvorlige bivirkninger, men kan være forbundet med kortvarige influenza lignende symptomer, der forsvinder i løbet af få timer. Længerevarende behandling kan være forbundet med øget risiko for blodpropper, forhøjet blodtryk og jernmangel. Protokol svarende til vores Epo behandlingsregime er tidligere blevet godkendt af Etisk Komite i København og Frederiksberg (Lundb*y et a*l., 2007;Thomse*n et a*l., 2007;Born*o et a*l., 2010;Jue*l et a*l., 2007;Lundb*y et a*l., 2008), og en øgning i hæmatokritten fra 45% til 49% (p<0.05) er fundet (Jue*l et a*l., 2007). Risikoen for blodpropper er større, hvis man samtidig har hypertension. Forsøgspersonerne vil kun blive inkluderet, hvis deres blodtryk er normalt (<135/85), og de vil blive ekskluderet, hvis hæmatokritten overstiger 55% (den nuværende doping grænse ligger på 50%, og grænsen for venesectio på 55%). For at undgå jernmangel vil alle forsøgsdeltagere blive behandlet med 100 mg jern p.o dagligt fra 1 uge før starten på forsøget indtil afslutningen.

Ved biopsi er der en meget lille risiko for infektion eller blødning i vævet. Forsøgspersonen instrueres i at henvende sig hos forsøgslederen ved symptomer herpå (rødme, hævelse, varme og ømhed). Derudover kan man opleve smerte fra biopsistedet de næste par dage, svarende til et ”trælår”. Der er en minimal risiko for at beskadige de små nerver i huden, hvilket vil medføre følelsesløshed i et mindre område på huden. Følesansen kommer oftest tilbage efter kortere eller sjældent længere tid. Vi har desuden haft to tilfælde af beskadigelse på en motorisknerve i selve musklen, som har medført lokal muskelatrofi uden påvirkning af muskelfunktionen.

Ved anlæggelse af intravenøse katetre (venflons) til blodprøvetagning og infusion er der en meget lille risiko for infektion og forsøgspersonerne instrueres i at henvende sig til forsøgslederen ved rødme og ømhed svarende til indstiksstederne.

Der er en lille risiko for hypoglykæmi under og efter den hyperinsulinemiske euglykæmiske clamp. Denne søges minimeret ved, at clampen styres af erfaren laborant og stabilt blodsukker etableres før forsøgspersonen forlader forskningslaboratoriet. Herudover udstyres forsøgspersonen med information om symptomer på hypoglykæmi samt druesukker til indtagelse i dette tilfælde.

Alvorlig hændelse eller alvorlig bivirkning: en hændelse eller bivirkning, som uanset dosis resulterer i død, er livstruende, medfører hospitalsindlæggelse eller forlængelse af hospitalsophold, resulterer i betydelig eller vedvarende invaliditet eller uarbejdsdygtighed. Hvis der optræder alvorlige hændelser/bivirkninger indsendes en liste til Lægemiddelstyrelsen og Videnskabsetisk Komité. I tilfælde af alvorlig hændelse/bivirkning stoppes forsøget øjeblikkeligt og evt. påkrævet behandling institueres. Det planlægges endvidere at følge forsøgspersonen indtil symptomfrihed og/eller at tilstanden er stationær.

Alle bivirkninger/hændelser vil endvidere fremgå af den afsluttende rapport.

Komplikationer forventes at kunne registreres i forbindelse med ovennævnte lægelige objektive observationer og behandles på forsøgslaboratoriet, idet forsøget udføres på et højt specialiseret sygehus.

Det samlede blodtab ved hver undersøgelsesdag andrager ca. 320 ml. Der er 10 uger mellem hver undersøgelsesrunde, så dette blodtab forventes ikke at inducere symptomer. Til sammenligning andrager en almindelig bloddonation ca. 500 ml. Herudover vil der blive opsamlet ca. 5 ml blod på dag 8, 15, 21, 36, 50, 64, 71 under behandlingen, samt på dag 78 og 92 under udvaskningsperioden. Disse vil blive brugt til at monitorer de hæmatologiske værdier. Desuden vil deltageren afgive 60 ml blod til mærkning af VLDL 1 uge før de metaboliske undersøgelsesdage. Blodtab i alt gennem undersøgelsen bliver således ca. 850 ml over ca. 4 måneder.

**Stråling:**

Den langsigtede risiko ved deltagelsen for forsøgspersonerne er relateret til strålebelastningen, der kan opgøres som følger:

I forbindelse med måling af glukose-omsætning infunderes der i alt 0,2 mSv og til bestemmelse af FFA turnover 0,4 mSv palmitattracer pr. metabolisk undersøgelsesdag. De infunderede isotoper til måling af carbamid og aminosyre omsætningen er stabile og udgør således ikke nogen risiko for deltageren.

Den samlede strålebelastning ved forsøgsdagene er således ca. 1,2mSv., hvilket er noget mindre, end den stråling man normalt modtager i løbet af et år (baggrundsstrålingen, som er ca. 3 mSv/år) og mindre end dosisgrænsen (20 mSv pr år for arbejdstagere over 18 år). DEXA skanning er hverken forbundet med smerte eller ubehag. I forbindelse med undersøgelsen får patienten en stråledosis på 0,1 mSv pr. gang sv.t. 1/30 af den årlige baggrundsstråling i Danmark og 1/10 af stråledosis ved et røntgenbillede af lungerne. Man kan teoretisk beregne den samlede ekstra risiko ved strålingen til en 0,014 % forøget risiko for pådragelse af en kræftlidelse i løbet af ens livstid. En gennemsnitsdansker vil altså forøge sin risiko fra 25,000 % til 25,014 %.

**Statistik**

Gruppesammenligninger vil blive foretaget med standard statistiske metoder (t-test og ANOVA eller tilsvarende non-parametrisk test). Inden for grupperne vil parret t-test eller tilsvarende non-parametrisk test blive anvendt. P værdi under 0,05 antages som signifikant resultat.

**Kildedata**.

Fra biopsierne: Protein niveauer målt med Western Blot og mRNA niveauer ved PCR, fibertype bestemmelse og aktivering af satellitceller målt ved immunhistokemi, fedtcellestørrelse samt aktivitetsassay for bl.a. LPL.

Fra blodprøverne: EPO, totalt, frit og bioaktivt IGF-I, IGFBP-1, glukose, insulin, C-peptid, frie fede syrer, adiponectin, metabolitter, glukagon, kortisol, katekolaminer, albumin, phenylalanin enrichment, glukose specifik aktivitet, glycerol, laktat, ALAT, basisk fosfatase, bilirubin, protrombin index, carbamid, VLDL, triglycerid, VLDL-TG, Proteomics analyser mm.

**Kvalitetskontrol og kvalitetssikring**

Investigator og de videnskabsetiske komitéer eller tilsvarende myndighed har adgang til at kontrollere de relevante data. Undersøgelserne følger GCP-principperne (Good Clinical Practice).

**Etiske overvejelser**

**Generelt**

Det tilkendegives hermed, at forsøget udføres i overensstemmelse med protokollen og gældende myndighedskrav. Oplysninger om forsøgspersonerne beskyttes efter lov om behandling af personoplysninger og sundhedsloven. Undersøgelsens start og gennemførelse forudsætter således godkendelse fra den Videnskabsetiske Komite i Region Midtjylland. Undersøgelsen vil blive anmeldt til Datatilsynet, Clinical trails Gov. og om nødvendigt til Lægemiddelstyrelsen. Undersøgelsen vil blive udført i overensstemmelse med Helsingfors-deklarationen II.

**Retningslinier for skriftlig og mundtlig information**

Den mundtlige og skriftlige information gives i overensstemmelse med Forskningsstyrelsens ”Vejledning om information og samtykke ved inddragelse af forsøgspersoner i biomedicinske forskningsprojekter”.

De potentielle forsøgspersoner kan rette henvendelse til investigator, og der vil blive aftalt tid og sted for mundtlig information. Den skriftlige information vil blive tilsendt på mail eller brev, i god tid, så forsøgspersonerne har mulighed for at gennemlæse materialet, inden den mundtlige information gives. Der vil blive informeret om muligheden for at medbringe bisidder til samtalen. Den mundtlige information vil blive givet af cand. scient (humanbiologi) Britt Christensen eller læge Birgitte Nellemann. Den mundtlige information om undersøgelsen vil foregå i enerum.

Der vil være mulighed for at stille spørgsmål, for at frasige sig viden om egen helbredstilstand og pjecen ”Dine rettigheder som forsøgsperson i et biomedicinsk forskningsprojekt” udgivet af Den Centrale Videnskabsetiske Komité vil blive udleveret. Det vil blive understreget, at deltagelsen er frivillig, og at tilsagn om deltagelse derfor kan trækkes tilbage på ethvert tidspunkt uden, at dette vil påvirke læge-patientforholdet.

Efter informationssamtalen vil der være mindst 1 døgns betænkningstid inden indhentelse af skriftlig samtykke på ”Standardsamtykkeerklæring”.

**Undersøgelsens forudsigelige risici og ulemper**

Anlæggelse af venekatetre er forbundet med lette smerter, og der er en beskeden risiko for infektion og blodansamling ved indstiksstedet. Der anvendes almindelig steril teknik.

Det samlede blodtab ved hver undersøgelsesdag andrager ca. 320 ml. Der er 10 uger mellem hver undersøgelsesrunde, så dette blodtab forventes ikke at inducere symptomer. Til sammenligning kan nævnes, at en almindelig bloddonation andrager ca. 500 ml.

Biopsier er forbundet med et vist ubehag, men foregår under lokalbedøvelse.

Alle metoder er velkendte i forsøgslaboratoriet og af de projektansvarlige, og vurderes til at være absolut acceptable i relation til det overordnede formål.

Den samlede stråledosis for deltagerne er 1,2 mSv ved hver af tracer forsøgene, hvilket svarer til en røntgenundersøgelse af bækkenet. Denne stråledosis resulterer i en 0,012 % forøget risiko for pådragelse af en kræftlidelse i løbet af ens livstid.

DEXA skanningen er hverken forbundet med smerte eller ubehag. I forbindelse med undersøgelsen får patienten en stråledosis på 0,1 mSv pr. gang sv.t. 1/30 af den årlige baggrundsstråling i Danmark og 1/10 af stråledosis ved et røntgenbillede af lungerne.

Denne samlede strålingsdosis (tracer + DEXA) øger på længere sigt kræftrisikoen med i alt 0,014 %. Livstidsrisikoen for kræft i forbindelse med deltagelse i undersøgelsen stiger således fra ca. 25 % til ca. 25,014 %.

**Fordele ved at deltage i undersøgelsen**

Forsøgspersonerne kan efter projektets afslutning få oplyst resultater af egne undersøgelser og få svar på blodprøver. De forsøgspersoner der randomiseres til træningsgruppen vil desuden indgå i et 12 ugers superviseret træningsforløb. Herudover vurderes det, at der ikke er andre umiddelbare fordele for den enkelte ved at deltage i undersøgelsen.

**Biobank**

Blod- og vævsprøver forventes anvendt ved analyserne og eventuelt overskydende væv destrueres herefter. Der oprettes således ikke en biobank, men en forskningsbiobank. Blodprøver vil blive sendt til USA, hvor proteomics analyserne vil blive udført. Prøverne destrueres her hvis der er mere tilbage efter at analyserne er færdige, og sendes således ikke retur til Danmark. Alle analyser forventes udført ved afslutningen af 2014.

**Håndtering og arkivering af data**

I forbindelse med indtastning af data forsøges fejl minimeret ved dobbeltindtastning og efterfølgende korrektur. Data opbevares i anonymiseret form. Data arkiveres på afdeling M, Århus Sygehus. Data skal arkiveres i 15 år efter undersøgelsens afslutning.

**Finansiering og forsikring**

Finansiering: Projektet finansieres af Medicinsk Endokrinologisk Afdeling, MEA og via en fondsbevilling fra World Anti Doping Agency (WADA) (200.000 US$). Der søges herudover om forskningsstøtte fra private fonde. Investigator Britt Christensen er ansat som fondslønnet post doc. på Århus Sygehus. Lønudgifter afholdes af Medicinsk Endokrinologisk Afdeling, MEA og WADA. Ph.d. studerende Birgitte Nellemann er aflønnet ved et samfinansieret stipendiat fra Århus Universitet.

Deltagere er dækket i overensstemmelse med lov om klage- og erstatningsadgang indenfor sundhedsvæsenet.

**Rapport**

Efter forsøgets afslutning udformes flere engelsksprogede artikler med henblik på publikation i videnskabelige tidsskrifter. Såvel positive som negative resultater offentliggøres.

**Kompensation**

Deltagerne i undersøgelsen vil modtage en ulempegodtgørelse på 5500 kr for træningsgruppen og 4500 kr for kontrolgruppen, for ulempe der er forbundet med forsøget. Transporten til og fra de to metaboliske dage vil blive godtgjort, det resterende står forsøgspersonerne selv for. Godtgørelsen er skattepligtig. Såfremt en deltager skulle udgå af studiet vil vedkommende få udbetalt kompensation i henhold til deltagelsestiden.

**Identifikation af nye serum markører til detektion af misbrug med erythropoietin**

Navn:..............................................................................................

**Samtykkeerklæring:**

"Jeg bekræfter hermed, at jeg efter at have modtaget information, såvel mundtligt som skriftligt, indvilliger i at deltage i det beskrevne videnskabelige forsøg.

Jeg er informeret om, at det er frivilligt at deltage, og at jeg når som helst og uden begrundelse kan trække mit tilsagn om deltagelse tilbage, uden at dette på nogen måde vil påvirke den nuværende eller fremtidige behandling af mig."

Dato:........................

Underskrift: (forsøgsdeltager)…………………………………………………………

Jeg giver samtykke til, at mit biologiske materiale må opbevares in en forskningsbiobank.

Jeg ønsker at få oplysninger om forsøgets resultater og eventuelle konsekvenser for mig.

Sæt ring: JA NEJ

Dato:…………………………

Underskrift (forsøgsdeltager):………………………………………………………

Med venlig hilsen

**Cand. Scient, Britt Christensen (forsøgs ansvarlig), Læge Birgitte Nellemann og Overlæge, Jens Otto Lunde Jørgensen (klinisk ansvarlig)**

**Medicinsk Endokrinologisk Afdeling, MEA , Århus Sygehus**

Forbeholdt den projektansvarlige

”Hermed attesteres det, at skriftlig deltagerinformation er blevet udleveret, samt at mundtlig information har fundet

sted:

Dato:__________________ Forsøgsansvarliges underskrift:________________________________

Lægmandsbeskrivelse

**Identifikation af nye serum markører til detektion af misbrug med erythropoietin**

Britt Christensen, Birgitte Nellemann, Jens Otto Lunde Jørgensen

Medicinsk Endokrinologisk Afdeling, MEA og Medicinsk Forskningslaboratorium, Århus Universitetshospital, Århus Sygehus, Nørrebrogade 44, DK-8000 Århus C

Tlf.: +45 89 49 20 35

**Samarbejdspartnere**

- Medicinsk Forskningslaboratorium

Århus Sygehus
Nørrebrogade 44
8000 Århus C.

- Medicinsk Endokrinologisk Afdeling

Århus Sygehus
Nørrebrogade 44
8000 Århus C.

- Institut for Idræt

Århus Universitet

Dalgas Avenue 4

8000 Århus C

**Tidsplan**

Forventet start: Juni 2011

Forventet afsluttet: August 2012

Sted

Medicinsk Forskningslaboratorium, afdeling M, Århus Sygehus.

Institut for Idræt, Århus Universitet

**Baggrund**

Erytropoietin (EPO) dannes i nyrerne, når ilttrykket i blodet bliver for lavt. EPO øger nydannelsen af røde blodlegemer i knoglemarven. EPO benyttes i dag som lægemiddel ved nyre- og kræftsygdomme, der medfører mangel på røde blodlegemer.

EPO øger produktionen af røde blodlegemer og dermed også mængden af ilt, der kan transporteres rundt i kroppen. Det er vist, at længerevarende brug af syntetisk EPO (rHuEPO) medføre, at man kan udføre det samme fysiske arbejde i længere tid, derfor bliver det til stadighed misbrugt af atleter specielt i udholdenhedsidrætter. Dette sker på trods af, at rHuEPO kom på anti-doping listen allerede i 1990. Det er derfor vigtigt at finde en god metode til at måle dette misbrug. Ikke kun for at sikre en fair konkurrence, men også for at mindske misbruget og dermed de bivirkninger dette medføre, såsom øget risiko for blodpropper.

Den eksisterende metode til at måle misbrug med rHuEPO, der er godkendt af det internationale anti-doping agentur (WADA), er baseret på forskelle i sukkergrupper bundet til henholdsvis rHuEPO, og det EPO vi selv danner i kroppen. Om denne metode vil være effektiv mod nye syntetiske EPO produkter er stadig uvist. Fremtidige EPO tests bør derfor også være baseret på biologiske markører, der ændres, når EPO niveauet stiger. Man har allerede undersøgt forskellige blodmarkører relateret til mængden af røde blodceller, men disse metoder har vist sig at være meget konservative, idet grænserne for normalområdet er meget brede. Dette for at undgå falsk-positive resultater.

Proteomics er en metode, hvormed man kan kigge på alle proteinerne i blodet på en gang. Også proteiner der er blevet ændret ganske lidt, kan man skelne fra hinanden. Hovedformålet med dette projekt er at undersøge effekten af længerevarende behandling med rHuEPO på ændringer i blodets proteiner hos raske unge mænd. Vi har i et tidligere studie med 16 dages behandling med rHuEPO vist, at dette medførte ændringer i blodets proteiner. Det er håbet, at nogen af disse proteiner kan bruges som markør for EPO misbrug i fremtiden. Vi ønsker ydermere at undersøge effekten af fysisk aktivitet på disse proteiner, idet idrætsudøvere jo alle er fysisk aktive. Effekten af fysisk aktivitet i sig selv er derfor vigtigt at få belyst, hvis disse proteiner skal bruges i en fremtidig anti-doping test.

EPO receptorer findes i mange forskellige typer væv ud over knoglemarven, for eksempel forskellige celler i hjernen, kræft celler, celler i tarmen og ikke mindst muskel væv, men effekten af EPO i disse væv mangler dog stadig at blive fuldt belyst.

Vi ønsker desuden at undersøge, hvilke effekter EPO har på fedt og muskelvæv, samt eventuelle effekter på omsætningen af fedt, sukker og protein.

**Formål**

At finde nye biomarkører i blodet der kan bruges til at detektere misbrug med EPO. Herudover at undersøge effekten af 10 ugers træning og behandling med EPO på en lang række andre parametre, såsom omsætningen af fedt, sukker og protein, samt ændringer i selve muskelvævet.

**Undersøgelsesplan**

I undersøgelsen vil indgå 40 raske unge mænd, disse rekrutteres via www.forsøgsperson.dk og diverse opslag på Århus universitet.

**Inklusionskriterier:**

- Skriftligt samtykke før undersøgelsens start
- Myndige, raske mænd
- Alder > 18 år og < 35 år
- Normalvægtige (BMI: 18-27)
- Utrænede (<2 timers fysisk aktivitet per uge, samt kondital under 45)
- Ikke ryger

**Eksklusions kriterier:**

- Kronisk sygdom
- Blodtryk over 135/85
- Hæmatokrit over 45%

**Design:**

- Forsøgspersonerne vil ved lodtrækning blive opdelt i følgende fire grupper (10 personer i hver gruppe)
  - Placebo behandling
  - Placebo behandling + udholdenhedstræning
  - rHuEPO behandling
  - rHuEPO behandling + udholdenhedstræning
- Forsøgspersonerne undersøges før og efter 10 uger EPO/placebo behandling og igen 3 uger efter behandlingens ophør.
- EPO (≈50 IU/kg)/placebo (saltvand) indgives s.c. 2 gange om ugen de første 3 uger i dosis 2x40 μg, og en gang i ugen fra og med uge 4-10 i dosis 20 μg.
- Udholdenhedstræningen vil bestå af 1-1.5 times cykling ved 65-80% af maksimal ilt optagelse.

**
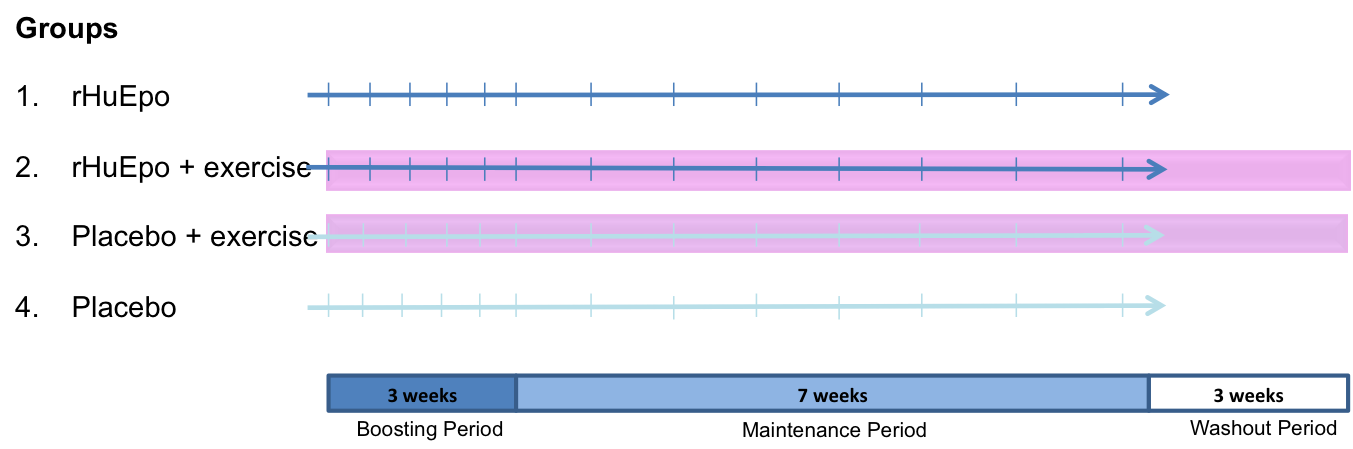
**

**Undersøgelses program:**

- Omsætningen af fedt, sukker og protein, muskel- og fedtbiopsier, blodprøver, maksimal iltoptagelsestest, DEXA skanning, MR skanning

**Primære end points:** Proteomics analyser på blodprøver

**Sekundære end points:** Omsætningen af fedt, sukker og proteiner, ændringer i muskelvæv

**Deltagerne vil udgå af undersøgelsen hvis:**

- De selv ønsker det
- Investigator skønner at deltageren ikke kan leve op til forsøgsprocedurerne eller pga. sikkerhedsmæssige årsager
- Deltageren udvikler alvorlige eller intolerable bivirkninger. I så fald vil forsøget for den enkelte deltager blive stoppet og investigator vil sørge for relevant opfølgning herunder henvisning til relevant behandling.

Deltageren må trække sig ud af forsøget på et hvilket som helst tidspunkt.

Data opnået fra udgået forsøgsperson vil blive anvendt, så vidt det er muligt – det vil afhænge af, hvornår i undersøgelsen en given forsøgsperson måtte udgå. De data, det er muligt at analysere sig frem til, vil blive benyttet.

**Metoder**

**Almindelig lægelig helbredsundersøgelse:** Foretages inden patienterne starter forsøgsperioden, der vil samtidig blive foretaget rutine blodprøver, DEXA skanning og konditallet vil blive målt.

**Metabolisk profil:** Undersøgelsen tager ca. 8 timer og vil blive fortaget før behandlingsforløbet og igen efter de 10 ugers behandling. Forsøgspersonen skal være fastende og ligge i en seng.

- - **Undersøgelse for substratoptagelse i underarm:** Der lægges 3 tynde plastik katetre (venflons) i 3 forskellige blodårer til indsprøjtning af forskellige stoffer samt til at tage blodprøver fra. Herved kan vi måle omsætningen af sukker, fedt og proteiner.
  - **Biopsi:** *Fedtbiopsi:* Fedtbiopsien tages fra maven under lokalbedøvelse. *Muskelbiopsi:* En muskelbiopsi tages fra lårmusklen under lokal bedøvelse
  - **Blodprøver:** Der tages blodprøver ca. hvert 20. minut under undersøgelsesdagen. Samlet blodtab andrager ca. 320 ml.
  - **Hyperinsulinæmisk euglykæmisk clamp:** Efter ca. 4 timers undersøgelse indsprøjter vi insulin i 2 timer for at måle insulin følsomheden, imens holdes blodsukker niveauet konstant.
  - **Indirekte kalorimetri:** Udåndingsluften analyseres 30 min før og efter den overfor beskrevne insulin stimulation. Dette giver mulighed for at beregne total energi forbrug, samt sukker, protein og fedt omsætningen.
  - **MR skanning:** Skanningstype til bestemmelse af fedtindhold i forskellige væv og organer. Patienten skal ligge helt stille og køres ind i scanneren som er et rør. Forsøgsdeltagerne vil blive orienteret om MR-skanninger - om at det er en ufarlig teknik uden nogen strålingsrisiko. Der vil være et højt lydniveau i skannerrummet. Personerne vil blive forsynet med lyddæmpende ørepropper.

**Blodprøver:** Der vil blive taget blodprøver før starten på behandlingen, på dag 8, 15, 21, 36, 50, 64, 71 samt på dag 78 og 92 under udvaskningsperioden. På disse dage vil blodets sammensætning blive analyseret og hvis værdierne bliver unormale, vil behandlingen blive afbrudt. Derudover vil der blive lavet proteomics analyser på blodprøver taget på følgende dage, før starten på studiet, på dag 21, 71 og 92.

**Blodtryksmålinger:** Blodtrykket vil blive målt på de samme dage, som der tages blodprøver.

Blod- og vævsprøver forventes anvendt ved analyserne og destrueres herefter. Der oprettes således ikke en biobank.

**Sikkerhedsvurdering**

Inden selve undersøgelsesdagene vil der blive foretaget en almindelig lægelig helbredsundersøgelse.

Der vil under hele forsøget være en læge og bioanalytiker tilstede, der kan foretage en almenvurdering af forsøgspersonen og foretage måling af vitale parametre (blodtryk, puls, blodsukker og bevidsthedsniveau).

Akut behandling med EPO er ikke forbundet med alvorlige bivirkninger, men kan være forbundet med kortvarige influenza lignende symptomer der forsvinder i løbet af få timer. Længerevarende behandling kan være forbundet med øget risiko for blodpropper, forhøjet blodtryk og jernmangel. En protokol svarende til det aktuelle EPO behandlings regime er tidligere blevet godkendt. I dette studie så man en øgning i hæmatokritten fra 45% til 49% (Jue*l et a*l., 2007). Risikoen for blodpropper er større, hvis man samtidig har forhøjet blodtryk. Forsøgspersonerne vil kun blive inkluderet, hvis deres blodtryk er normalt (<135/85), og de vil blive ekskluderet hvis hæmatokritten overstiger 55% (den nuværende doping grænse er 50% og grænsen for blodtapning på 55%). For at undgå jernmangel vil alle forsøgsdeltagerne blive behandlet med 100 mg jern tabletter dagligt fra 1 uge før behandlingens start, og til forsøget er afsluttet.

Ved udtagning af vævsprøver er der en meget lille risiko for betændelse eller blødning i vævet. Forsøgspersonen instrueres i at henvende sig hos forsøgslederen ved symptomer herpå (rødme, hævelse, varme og ømhed). Derudover kan man opleve smerte fra biopsi stedet de næste par dage. Der er en minimal risiko for at beskadige de små nerver i huden, hvilket vil medføre følelsesløshed i et mindre område på huden. Følesansen kommer som oftest tilbage efter kortere eller sjældent længere tid. Vi har desuden haft to tilfælde af beskadigelse på en nerve i selve musklen, som har medført lokal muskelsvind, dog uden nogen påvirkning af muskelfunktionen.

Ved anlæggelse af tynde plastikrør (venflons) i blodkar til blodprøvetagning og til indsprøjtning er der en meget lille risiko for betændelse og forsøgspersonerne instrueres i at henvende sig til forsøgslederen ved rødme og ømhed svarende til indstiksstederne.

Der er en lille risiko for at udvikle lavt blodsukker under og efter den såkaldte insulin stimulation. Denne søges minimeret ved, at clampen styres af erfaren laborant, og blodsukkeret normaliseres før forsøgspersonen sendes hjem. Herudover udstyres forsøgspersonen med information om symptomer på for lavt blodsukker samt druesukker til indtagelse i dette tilfælde.

Alvorlig hændelse eller alvorlig bivirkning: en hændelse eller bivirkning, som uanset dosis resulterer i død, er livstruende, medfører hospitalsindlæggelse eller forlængelse af hospitalsophold, resulterer i betydelig eller vedvarende invaliditet eller uarbejdsdygtighed. Hvis der optræder alvorlige hændelser/bivirkninger indsendes en liste til Lægemiddelstyrelsen og Videnskabsetisk Komité. I tilfælde af alvorlig hændelse/bivirkning stoppes forsøget øjeblikkeligt og evt. påkrævet behandling institueres. Det planlægges endvidere at følge forsøgspersonen indtil symptomfrihed og/eller at tilstanden er stationær.

Alle bivirkninger/hændelser vil endvidere fremgå af den afsluttende rapport.

Komplikationer forventes at kunne registreres i forbindelse med ovennævnte lægelige objektive observationer og behandles på forsøgslaboratoriet, idet forsøget udføres på et højt specialiseret sygehus.

Det samlede blodtab ved hver undersøgelsesdag andrager ca. 320 ml. Der er min. 10 uger mellem hver undersøgelsesrunde, så dette blodtab forventes ikke at give symptomer. Til sammenligning kan nævnes, at en almindelig bloddonation andrager ca. 500 ml. Der vil blive opsamlet 60 ml blod 1 uge forud for hver undersøgelsesdag, med henblik på VLDL undersøgelser. Desuden vil der under studiet blive opsamlet ca. 5ml blod på følgende dage;8, 15, 21, 36, 50, 64, 71, 78 og 92. Dette gøres med henblik på at monitorer blod værdierne.

**Stråling:**

Den samlede strålebelastning er ca. 1,4 mSv., hvilket er noget mindre, end den stråling man normalt modtager i løbet af et år (baggrundsstrålingen, som er ca. 3 mSv/år) og mindre end dosisgrænsen (20 mSv pr år for arbejdstagere over 18 år). Man kan teoretisk beregne den ekstra risiko ved strålingen til 0,014 % forøget risiko for pådragelse af en kræftlidelse i løbet af ens livstid. En gennemsnitsdansker vil altså forøge sin risiko fra 25,000 % til 25,014 %.

**Statistik**

Der vil blive anvendt standard statistiske metoder.

**Adgang til kildedata**

Investigator og de videnskabsetiske komitéer eller tilsvarende myndighed har adgang til at kontrollere de relevante data.

**Etiske overvejelser**

**Generelt**

Det tilkendegives hermed, at forsøget udføres i overensstemmelse med protokollen og gældende myndighedskrav. Undersøgelsens start og gennemførelse forudsætter således godkendelse fra den Videnskabsetiske Komite i Region Midtjylland. Undersøgelsen vil blive anmeldt til Datatilsynet, Clinical Trials Gov. og om nødvendigt til Lægemiddelstyrrelsen. Undersøgelsen vil blive udført i overensstemmelse med Helsingfors-deklarationen II.

**Retningslinier for skriftlig og mundtlig information**

Den mundtlige og skriftlige information gives i overensstemmelse med Forskningsstyrelsens Vejledning om information og samtykke ved inddragelse af forsøgspersoner i biomedicinske forskningsprojekter.

De potentielle forsøgspersoner kan rette henvendelse til investigator, og der vil blive aftalt tid og sted for mundtlig information. Der vil blive informeret om muligheden for at medbringe bisidder til samtalen. Den mundtlige information vil blive givet af cand. scient (Humanbiologi) Britt Christensen eller læge Birgitte Nellemann. Den mundtlige information om undersøgelsen vil foregå i enerum.

Der vil være mulighed for at stille spørgsmål, for at frasige sig viden om egen helbredstilstand og pjecen ”Forsøgspersonens rettigheder i et biomedicinsk forskningsprojekt” udgivet af Den Centrale Videnskabsetiske Komité vil blive udleveret. Det vil blive understreget, at deltagelsen er frivillig og at tilsagn om deltagelse derfor kan trækkes tilbage på ethvert tidspunkt uden at dette vil påvirke læge-patientforholdet.

Efter informationssamtalen vil der være mindst 1 døgns betænkningstid inden indhentelse af skriftlig samtykke på ”Standardsamtykkeerklæring”.

**Undersøgelsens forudsigelige risici og ulemper**

Anlæggelse af små plastikkatetre i blodårer er forbundet med lette smerter, og der er en beskeden risiko for betændelse og blodansamling ved indstiksstedet. Der anvendes almindelig steril teknik.

Det samlede blodtab ved hver undersøgelsesdag andrager ca. 320 ml. Der er 10 uger mellem hver undersøgelsesrunde, så dette blodtab forventes ikke at give symptomer. Til sammenligning kan nævnes, at en almindelig bloddonation andrager ca. 500 ml.

Muskelprøvetagning er forbundet med et vist ubehag, men foregår under lokalbedøvelse.

Alle metoder er velkendte i forsøgslaboratoriet og af de projektansvarlige, og vurderes til at være absolut acceptable i relation til det overordnede formål.

Den samlede stråledosis for deltagerne er 1,4 mSv, hvilket svarer til mindre end en røntgenundersøgelse af bækkenet. Denne stråledosis resulterer i en 0,014 % forøget risiko for pådragelse af en kræftlidelse i løbet af ens livstid.

**Fordele ved at deltage i undersøgelsen**

Forsøgspersonerne kan efter projektets afslutning få oplyst resultater af egne undersøgelser og få svar på blodprøver. De forsøgspersoner der randomiseres til træningsgruppen vil desuden indgå i et 12 ugers overvåget træningsforløb. Men herudover vurderes det, at der ikke er andre umiddelbare fordele for den enkelte ved at deltage i undersøgelsen.

**Biobank**

Blod- og vævsprøver forventes anvendt ved analyserne og destrueres herefter. Der oprettes således ikke en biobank. Blodprøver vil blive sendt til USA, hvor proteomics analyserne vil blive udført. Prøverne destrueres, hvis der er mere tilbage, efter at analyserne er færdige.

**Håndtering og arkivering af data**

I forbindelse med indtastning af data forsøges fejl minimeret ved dobbeltindtastning og efterfølgende korrektur. Data opbevares i anonymiseret form. Data arkiveres på afdeling M, Århus Sygehus. Data arkiveres i 15 år efter undersøgelsens afslutning.

**Finansiering og forsikring**

Finansiering: Projektet finansieres af Medicinsk Afdeling MEA og World anti-doping agency (WADA). Der søges om yderligere forskningsstøtte fra private fonde. Investigator Britt Christensen er ansat som fondslønnet post doc. på Århus Sygehus. Lønudgifter afholdes af Medicinsk Afdeling MEA og WADA. Ph.d. studerende Birgitte Nellemann er aflønnet ved et samfinansieret stipendiat fra Århus Universitet.

Deltagere er dækket i overensstemmelse med lov om klage- og erstatningsadgang indenfor sundhedsvæsenet.

**Kompensation**

Deltagerne i undersøgelsen vil modtage en ulempegodtgørelse på 5500 kr for træningsgruppen og 4500 kr. for kontrolgruppen, for ulempe der er forbundet med forsøget. Transportudgifter i forbindelse med de 2 metaboliske dage vil blive dækket, det resterende står forsøgspersonerne selv for. Godtgørelsen er skattepligtig. Såfremt en deltager skulle udgå af studiet vil vedkommende få udbetalt kompensation i henhold til deltagelsestiden.

**Publikation**

Efter forsøgets afslutning udformes engelsksprogede artikler med henblik på publikation i videnskabelige tidsskrifter. Såvel positive som negative resultater offentliggøres.

Deltagerinformation

**Identifikation af nye serum markører til detektion af misbrug med erythropoietin**

**Du anmodes hermed om at deltage i et videnskabeligt projekt.**

**Formål**

At finde nye biomarkører i blodet der kan bruges til at detektere misbrug med EPO. Desuden at undersøge effekten af 10 uger træning og behandling med EPO på en lang række andre parametre, såsom omsætningen af fedt, sukker og protein, samt ændringer i selve muskel- og fedtvævet.

**EPO**

Erytropoietin (EPO) dannes i nyrerne, når ilt trykket i blodet bliver for lavt. EPO øger nydannelsen af røde blodlegemer i knoglemarven. EPO benyttes i dag som lægemiddel ved nyresygdomme, der medfører mangel på røde blodlegemer. Akut behandling med EPO er ikke forbundet med alvorlige bivirkninger, men kan være forbundet med kortvarige influenza lignende symptomer, der forsvinder i løbet af få timer. Længerevarende behandling kan være forbundet med øget risiko for blodpropper, forhøjet blodtryk og jernmangel. Blodets sammensætning og blodtrykket vil blive målt gennem hele studiet, og der vil blive givet jern tilskud for at undgå jernmangel.

EPO øger produktionen af røde blodlegemer og dermed også mængden af ilt, der kan transporteres rundt i kroppen. Det er vist, at længerevarende brug af syntetisk EPO (rHuEPO) medfører, at man kan udføre det samme fysiske arbejde i længere tid, derfor bliver det til stadighed misbrugt af atleter specielt i udholdenheds idrætter. Dette på trods af at rHuEPO kom på anti-doping listen allerede i 1990. Det er derfor vigtigt at finde en god metode til at måle dette misbrug. Ikke kun for at sikre en fair konkurrence, men også for at mindske misbruget og dermed de bivirkninger dette medføre. Vi ønsker med dette forsøg at finde nye markører i blodet, der kan bruges i en fremtidig anti-doping test.

EPO receptorer findes i mange forskellige typer væv ud over knoglemarven, f.eks. forskellige celler i hjernen, kræft celler, celler i tarmen og ikke mindst muskelvæv, men effekten af EPO i disse væv mangler dog stadig at blive fuldt belyst.

Vi ønsker sekundært at undersøge, hvilke effekter EPO har på fedt- og muskelvæv, samt eventuelle effekter på omsætningen af fedt, sukker og protein. Vi har i et tidligere akut studie med EPO vist, at der var tegn på ændringer i fedt omsætningen.

**Undersøgelsens opsætning**

Du er:

- Rask
- 18-35 år
- Normalvægtig (BMI: 18-27)
- Utrænet (<2 timer pr uge)
- Ikke ryger
- Ikke forhøjet blodtryk

Deltagelse indebærer:

- Screening: Samtale om undersøgelsen, almindelig lægelig undersøgelse der indebærer optagelse af sygdomsanamnese, måling af blodtryk, livvide, vægt, højde, hjertediagram og rutineblodprøve samt måling af kropssammensætningen ved en DEXA skanning, og en maksimal iltoptagelses test på cykel. Varighed ca. 3 timer.
- I alt 2 hele forsøgsdage af ca. 8 timers varighed før og efter et 10 ugers forløb. Samt MR skanning før og efter de 10 uger.
- Blodprøvetagning løbende under de 10 uger forsøget forløber og 3 uger herefter (i alt 13 uger)
- Du vil ved lodtrækning enten blive behandlet med EPO eller placebo (saltvand), der vil blive sprøjtet ind under huden af en af de til projektet tilknyttede personer. Du vil ikke få at vide, om du får EPO eller saltvand før forsøget er afsluttet.

Du vil således skulle møde på afdelingen til behandling (evt. i forbindelse med træningen), to gange om ugen de første 3 uger, og en gang om ugen de sidste 7 uger. Der vil desuden på nogle af disse dage blive taget en blodprøve.

- Du vil også ved lodtrækning komme enten i en kontrolgruppe eller i en træningsgruppe. Sidst nævnte skal træne i 1-1.5 time 3 gange om ugen i de 10 uger forsøget forløber. Træningen vil foregå på Institut for Idræt, Dalgas Avenue i Århus.

**Undersøgelses program (metabolisk profil):**

- Undersøgelsen tager ca. 8 timer, og vil på selve dagen være fastende og ligge i en seng.
  - **Undersøgelse for optagelse af forskellige stoffer i underarm:** Der lægges 3 tynde plastik katetre (venflons) i 3 forskellige blodårer til indsprøjtning af forskellige stoffer samt til at tage blodprøver fra. Herved kan vi måle omsætningen af fedt, sukker og proteiner.
  - **Biopsi:** Efter 1 og 5 timer tages en fedt- og muskelprøve. *Fedtbiopsi:* Fedtprøven tages fra maven under lokalbedøvelse. *Muskelbiopsi:* En muskelprøve tages fra lårmusklen under lokalbedøvelse, og kan efterfølgende føles som et ”trælår”.
  - **Blodprøver:** Der tages blodprøver ca. hvert 20. minut under undersøgelsesdagen. Samlet blodtab andrager ca. 320 ml.
  - **Insulin sensitivitet:** Insulin søger for at sukkeret i blodet bliver transporteret ind i bl.a. musklerne. Efter ca. 4 timers undersøgelse indsprøjter vi insulin i 2 timer for at måle insulinfølsomheden, imens holdes blodsukker niveauet konstant.
  - **Undersøgelse af udåndingsluften:** Udåndingsluften analyseres 30 min før og efter den overfor beskrevne insulin undersøgelse. Dette giver mulighed for at beregne det totale energiforbrug, samt omsætningen af sukker, protein og fedt.

Forhold der gør, at forsøgspersoner udelukkes fra videre deltagelse i forsøget

- Du selv ønsker det.
- Hvis den forsøgsansvarlige skønner, at du ikke kan leve op til forsøgsprocedurerne eller pga. sikkerhedsmæssige årsager.
- Hvis du udvikler alvorlige eller intolerable bivirkninger. I så fald vil forsøget blive stoppet og den forsøgsansvarlige vil foranledige relevant opfølgning herunder henvisning til relevant behandling, derefter vurderes det om forsøget som helhed skal bringes til ophør.

**Risici**

Anlæggelse af katetre, udtagning af vævsprøver, samt anlæggelse af lokalbedøvelse kan være forbundet med let ubehag samt en lille risiko for udvikling af blodansamling og betændelse ved indstiksstedet. Ved tegn på betændelse (rødme, varme, ømhed, hævelse) skal du henvende dig til den projektansvarlige (i dagtid) eller på skadestuen (i aften- og nattetid). Efter udtagelsen af muskelprøven, kan det de efterfølgende dage føles som et ”trælår”. Der er desuden lille risiko for at beskadige de små nerver i huden, dette vil i en periode medføre lokal følelsesløshed. Vi har herudover set to tilfælde af beskadigelse af en nerve i musklen, der har medført lokalt tab af muskelmasse, dog uden nogen påvirkning af muskelfunktionen. Det samlede blodtab i forbindelse med undersøgelsen vil ikke overstige 320 ml på hver forsøgsdag.

Der vil blive brugt radioaktive stoffer til undersøgelsen af omsætning af fedt, sukker og protein. Den samlede stråling (1,2 mSv) er noget mindre, end den man modtager på et år fra baggrundsstråling (3 mSv/år). Stråledosis svarer til mindre end en røntgenundersøgelse af bækkenet. Herudover vil hver DEXA skanning give en stråling på 0,1 mSv.

Man kan teoretisk beregne den ekstra risiko ved strålingen til en 0,014 % forøget risiko for pådragelse af en kræftlidelse i løbet af ens livstid. En gennemsnitsdansker vil altså forøge sin risiko fra 25,000 % til 25,014 %.

MR skanningen er ikke forbundet med nogen strålingsrisiko. Undersøgelsen kan være svær for personer med klaustrofobi, da man køres ind i et rør der er åbent i begge ender. Undersøgelsen er også forbundet med en del larm, men i vil blive udstyret med ørepropper.

Alle anvendte undersøgelsesmetoder har været anvendt som rutine på vores forsknings-laboratorium igennem flere år.

**Information**

Du vil blive orienteret såvel mundtligt som skriftligt om undersøgelsens formål og omfang, og om at du på et hvilket som helst tidspunkt kan tilbagetrække et givet tilsagn om deltagelse og udtræde af forsøget uden begrundelse herfor, og det vil hverken nu eller i fremtiden påvirke din behandling eller læge-patientforholdet.

Du har ret til at medbringe en bisidder til informationssamtalen, til at udbede betænkningstid og til at frasige dig viden om din egen helbredstilstand. I forbindelse med informationssamtalen vil du få udleveret pjecen ”Forsøgspersonens rettigheder i et biomedicinsk forskningsprojekt” udgivet af Den Centrale Videnskabsetiske Komité. Undersøgelsen vil blive stoppet, såfremt der måtte optræde alvorlige bivirkninger eller alvorlige hændelser.

Du vil blive yderligere informeret, hvis der fremkommer oplysninger, der er relevante for din fortsatte deltagelse i forsøget.

**Den mulige nytte ved forsøget**

I dag ved man nøje hvilken effekt EPO har på blodet og ilt transporten i blodet, men effekten andre steder i kroppen er meget dårligt belyst. EPO benyttes i dag som doping middel specielt i udholdenheds idrætter, og det er vores håb med dette forsøg at kunne finde nye markører, der kan bruges i en fremtidig anti-doping test. Herudover håber vi på, at undersøgelsen kan være med til at øge vores viden om effekten af EPO på muskelvæv, specielt er vi interesseret i at finde ud af om EPO har nogen effekt på musklens følsomhed for insulin, har nogen effekt på omsætningen af sukker, fedt og proteiner i musklerne, og om musklerne ændrer sammensætning i forbindelse med behandlingen. Umiddelbart er der intet udbytte for dig som deltager i undersøgelsen. Hvis du bliver udtrukket til at deltage i træningsgruppen, vil du dog modtage 10 ugers superviseret træning. Efter projektets afslutning kan du få oplyst resultater og få svar på blodprøver.

**Økonomi**

For deltagelse i undersøgelsen vil du modtage en ulempegodtgørelse på 5500 kr., hvis du kommer i træningsgruppen og 4500 kr. hvis du ikke skal træne, dette beløb dækker over ulempe der er forbundet med forsøget. Herudover dækker vi transportudgifter i forbindelse med de 2 metaboliske undersøgelsesdage, de resterende transportudgifter står du selv for. Godtgørelsen er skattepligtig. Såfremt du udtræder af forsøget før afslutningen vil du modtage kompensation i henhold til den tid du har deltaget.

Projektet finansieres af Medicinsk Endokrinologisk Afdeling, MEA og via en fondsbevilling fra World Anti Doping Agency (WADA) (200.000 US$).

**Godkendelse**

Projektet er godkendt af den Videnskabsetiske Komité for Region Midtjylland og anmeldt og godkendt af Datatilsynet.

**Data**

Projektet er anmeldt til Datatilsynet og følger de her gældende retningslinier. Anonymiseret data opbevares i 15 år efter afslutning af projektet. Persondata opbevares strengt fortrolige.

Oplysninger og informationer om dine helbredsforhold og private forhold er omfattet af tavshedspligt. Du har mulighed for at få aktindsigt.

Medarbejdere fra GCP-enheden og den lokale videnskabsetiske komité har adgang til fortegnelser over forsøgspersoner med henblik på at verificere procedurer og/eller data i forbindelse med forsøget.

**Klageadgang, erstatning og kompensation**

Det skal oplyses, at du har klageadgang samt mulighed for erstatning efter lov om klage- og erstatningsadgang indenfor sundhedsvæsenet.

**Kontaktpersoner**

Den praktiske del af projektet udføres af:

**Britt Christensen**

Telefon arbejde: 89 49 20 35

Mobiltelefon: 31 19 48 19

Email: [britt.christensen@ki.au.dk](mailto:britt.christensen@ki.au.dk)

**Birgitte Nellemann**

Telefon arbejde: 89 49 20 32

Mobiltelefon: 29 61 14 46

Email: bnso@svf.au.dk

Klinisk ansvarlig er:

**Overlæge Jens Otto Lunde Jørgensen**

**Medicinsk Afdeling M, Århus Sygehus**

**Mobiltelefon: 20 72 73 83**

I tilfælde af komplikationer (som beskrevet ovenfor) kan den vagthavende bagvagt på Medicinsk Afdeling MEA kontaktes: **89 49 33 33 kode 2067**

**Er du en rask, utrænet mand i alderen 18-35 år, og har du lyst til at medvirke i et videnskabeligt forsøg?**

**Formålet er at undersøge effekten af EPO og træning.**

For at kunne deltage:

- skal du være mand og **ikke** dyrke motion til dagligt
- du skal være normalvægtig med BMI 18-27
- du skal være 18-35 år
- du må ikke tage noget medicin

Undersøgelsen omfatter:

- Forundersøgelse med lægelig helbredsundersøgelse, undersøgelse af kropssammensætningen ved DEXA skanning, konditest, blodprøver.
- 2 forsøgsdage af 7-8 timers varighed adskilt af 3 måneder, på hver forsøgsdag vil der blive taget 2 fedt- og 2 muskelbiopsier, samt MR skanning.
- Ved lodtrækning udvælges du til
  - Enten 3 måneders superviseret cykel træning 3 gange om ugen på Institut for Idræt
  - Eller 3 måneder hvor du ikke ændre dit aktivitets niveau
- Ved lodtrækning udvælges du til
  - Enten ugentlige indsprøjtninger med EPO
  - Eller ugentlige indsprøjtninger med saltvand
- I løbet af de 3 måneder skal du have taget blodprøver løbende.

Forventet plan:

- Projektet starter i august 2011 og der vil være inklusion af forsøgspersoner i perioden august-november. Forsøget forløber herefter 3 måneder fra første forsøgsdag.
- Såfremt du kommer i træningsgruppen skal du kunne deltage i træning på Institut for Idræt 3 gange om uge i 3 måneder.

Før undersøgelsen vil du få skriftlig og mundtlig information.

Projektet er godkendt af den Videnskabsetiske Komite for Region Midtjylland. Der er opnået støtte til projektet fra World Anti Doping Agency (WADA).

Kommer du i træningsgruppen vil du modtage en skattepligtig ulempegodtgørelse på 5500 kr. eller svarende til varigheden af din deltagelse. Kommer du i kontrolgruppen vil du modtage en skattepligtig ulempegodtgørelse på 4500 kr. eller svarende til varigheden af din deltagelse.

Selve undersøgelserne foregår på:

Medicinsk Forskningslaboratorium

Medicinsk Endokrinologisk Afdeling

Aarhus Sygehus

Nørrebrogade 44, bygning 3

8000 Århus C

Træning foregår på:

Institut for Idræt

Dalgas Avenue 4

8000 Århus C

For yderligere information kontakt:

Britt Christensen Birgitte Nellemann

Cand Scient, PhD Læge, PhD studerende

Medicinsk Endokrinologisk Afdeling Medicinsk Endokrinologisk Afdeling

Århus Sygehus Århus Sygehus

Nørrebrogade 44, byg. 2 Nørrebrogade 44, byg. 2

8000 Århus C 8000 Århus C

Tlf: +45 8949 2035 Tlf: +45 8940 2032

E-mail: [britt.christensen@ki.au.dk](mailto:britt.christensen@ki.au.dk) E-mail: birgitte.nellemann@ki.au.dk

Reference List

Barroso O, Mazzoni I, & Rabin O (2008). Hormone abuse in sports: the antidoping perspective. *Asian J Androl* **10**, 391-402.

Borno A, Aachmann-Andersen NJ, Munch-Andersen T, Hulston CJ, & Lundby C (2010). Screening for recombinant human erythropoietin using [Hb], reticulocytes, the OFF(hr score), OFF ( z score) and Hb ( z score): status of the Blood Passport. *Eur J Appl Physiol* **109**, 1003-1005.

Catlin DH, Fitch KD, & Ljungqvist A (2008). Medicine and science in the fight against doping in sport. *J Intern Med* **264**, 99-114.

Christensen B, Sackmann-Sala L, Cruz-Topete D, Jorgensen JO, Jessen N, Lundby C, & Kopchick JJ (2010). Novel serum biomarkers for erythropoietin use in humans: A proteomic approach. *J Appl Physiol*.

Gore CJ, Parisotto R, Ashenden MJ, Stray-Gundersen J, Sharpe K, Hopkins W, Emslie KR, Howe C, Trout GJ, Kazlauskas R, & Hahn AG (2003). Second-generation blood tests to detect erythropoietin abuse by athletes. *Haematologica* **88**, 333-344.

Hojman P, Gissel H, & Gehl J (2007). Sensitive and precise regulation of haemoglobin after gene transfer of erythropoietin to muscle tissue using electroporation. *Gene Ther* **14**, 950-959.

Juel C, Thomsen JJ, Rentsch RL, & Lundby C (2007). Effects of prolonged recombinant human erythropoietin administration on muscle membrane transport systems and metabolic marker enzymes. *Eur J Appl Physiol* **102**, 41-44.

Lundby C, Hellsten Y, Jensen MB, Munch AS, & Pilegaard H (2008). Erythropoietin receptor in human skeletal muscle and the effects of acute and long term injections with recombinant human erythropoietin on the skeletal muscle. *J Appl Physiol* **104**, 1154-1160.

Lundby C & Robach P (2009). Assessment of total haemoglobin mass: can it detect erythropoietin-induced blood manipulations? *Eur J Appl Physiol* **108**, 197-200.

Lundby C, Thomsen JJ, Boushel R, Koskolou M, Warberg J, Calbet JA, & Robach P (2007). Erythropoietin treatment elevates haemoglobin concentration by increasing red cell volume and depressing plasma volume. *J Physiol* **578**, 309-314.

Midgley AW, McNaughton LR, & Wilkinson M (2006). Is there an optimal training intensity for enhancing the maximal oxygen uptake of distance runners?: empirical research findings, current opinions, physiological rationale and practical recommendations. *Sports Med* **36**, 117-132.

Pascual JA, Belalcazar V, de BC, Gutierrez R, Llop E, & Segura J (2004). Recombinant erythropoietin and analogues: a challenge for doping control. *Ther Drug Monit* **26**, 175-179.

Thomsen JJ, Rentsch RL, Robach P, Calbet JA, Boushel R, Rasmussen P, Juel C, & Lundby C (2007). Prolonged administration of recombinant human erythropoietin increases submaximal performance more than maximal aerobic capacity. *Eur J Appl Physiol* **101**, 481-486.
