# Supplementary material for: Serum Proteomic Changes after Randomized Prolonged Erythropoietin Treatment and/or Endurance Training: Detection of Novel Biomarkers
Source: PLoS One. 2015 Feb 13;10(2):e0117119. doi: 10.1371/journal.pone.0117119 (PMC4332672; doi:10.1371/journal.pone.0117119)
Supplement: S2 Protocol — (DOCX) [file pone.0117119.s003.docx]

Nr: M-20110035

**Identification of new serum markers for detection of abuse with erythropoietin**

Britt Christensen, Birgitte Nellemann, and Jens Otto Lunde Jørgensen

Department of Endocrinology and Internal Medicine, MEA, and Medical

Research Laboratory, Aarhus University Hospital,

Norrebrogade 44, DK-8000 Aarhus C

Tel.: +45 89 49 20 35

**INVOLVED PARTNERS**

• Medical Research Laboratory

Aarhus University Hospital

Norrebrogade 44

8000 Aarhus C

Denmark

• Department of Endocrinology and Internal Medicine

Aarhus University Hospital

Norrebrogade 44

8000 Aarhus C

Denmark

• Institute of Sport Science

Aarhus University

Dalgas Avenue 4

8000 Aarhus C

Denmark

**TIMETABLE**

Estimated start: June 2011

Expected completion: August 2012

**LOCATION**

Medical Research Laboratory, Aarhus University Hospital.

Institute of Sports Science, Aarhus University.

**BACKGROUND**

Erythropoietin (Epo) is a 34 kDa glycoprotein that is synthesized in the kidneys in response to low oxygen tension. Epo stimulates the formation of new erythrocytes in the bone marrow, while plasma volume is reduced (Lundby et al., 2007). This eventually increases the oxygen-binding capacity in blood.

Recombinant human Epo (rHuEpo) first came on the market in 1989, and is now used to treat uremic and cancer-induced anemia. Because Epo stimulates red blood cell production, and injection of rHuEpo for a prolonged period increases the aerobic capacity by more than 50% (Thomsen et al., 2007), it will continue to be abused by athletes in endurance sports (Barroso et al. 2008; Pascual et al. 2004; Catlin et al., 2008). This despite the fact that rHuEpo was prohibited by IOC already in 1990. It is therefore extremely important to find a sensitive and robust method to detect such abuse. Not only to ensure a fair competition but also to reduce the abuse and the risk of side effects, including increased risk for cardiovascular disease. The existing method for detecting rHuEpo, approved by WADA, is based on differences in the glycosylation degree and pattern between rHuEpo and endogenous Epo (Catlin et al. 2008; Pascual et al., 2004). However, this method is insensitive and relatively expensive (Pascual et al., 2004; Gore et al., 2003). Whether this approach will be effective against new generations of Epo derivatives produced in human cell lines, and thus expresses the same glycosylation degree and pattern as rHuEpo, is still unknown (Gore et al.

2003). In addition, gene doping is also a future drug strategy one will have to deal with. Stable transfection of the Epo gene in tissue has already been shown in animal models (Hojman et al., 2007). Gene doping was therefore in 2003 added to WADA and the IOC's list of "Prohibited Substances and Methods in Sport"(Pascual et al., 2004). This stresses the importance of developing new and more sensitive Epo analysis. Thus, future anti-doping tests should not only focus on detecting rHuEpo and its derivatives, but also on biological markers that change by Epo exposure. Such markers have been studied. Total hemoglobin mass and reticulocyte percentage (_OFFhr-score_) and the so-called blood-passport (Gore et al., 2003) have been suggested as markers for Epo abuse. The problem with the OFF_hr-score_ is that the cut-off values are based on the average population level, and because of large inter-individual differences the cut-off values (upper and lower limit) are very broad, in order to avoid false-positive results. This ultimately leads to a low detection level (Lundby & Robach, 2009; Borno et al., 2010). Also the blood pass, where one follows the athletes’ blood values over time, proved not to guarantee a drug free sport (Borno et al., 2010).

Proteomics has proven to be a good way to characterize all proteins expressed in a biological system, such as blood. We have already in a previous study showed that 16 days of treatment with rHuEpo resulted in significant changes in several serum proteins (Christensen et al.

2010). The main objective of this project is to investigate the effect of prolonged rHuEpo treatment on changes in the serum proteome of healthy young men. Furthermore, we want to investigate the effect of rHuEpo and physical activity on these markers, as it is important to elucidate the effect of physical activity, if these markers should be used as anti-doping markers in the future.

Epo receptors are found in a variety of different types of tissues other than the bone marrow, including neurons, astrocytes, microglia, cancer cells, gastric mucosal cells and especially muscle tissue. The effect of Epo in these tissues still needs to be fully elucidated.

Furthermore, in this study we also want to look at the effects Epo has on fat and muscle tissue and possible effects on substrate metabolism and insulin sensitivity. In a previous study, we have found that rHuEpo acutely might have an effect on fat metabolism (unpublished data). The study also showed that Epo receptors are found in muscle tissue and that prolonged treatment with rHuEpo results in changes in the muscle proteome. These results suggest that Epo may induce a shift in muscle fiber type from a fast glycolytic phenotype to a slower phenotype (data submitted).

**PURPOSE**

To identify new serum biomarkers to detect abuse of rHuEpo. Secondarily, we want to examine the effects of 10 weeks of rHuEpo treatment and training on a wide range of other parameters e.g. turnover of fat, carbohydrate, and protein, muscle type changes, and activation of satellite cells in skeletal muscle.

**DESIGN**

• The experiment is a single-blind, randomized study.

• 40 subjects in total will be included and randomly assigned to one of the following groups (n = 10);

o Placebo treatment

o Placebo treatment + endurance training

o rHuEpo treatment

o rHuEpo treatment + endurance training

• Subjects are examined before and after 10 weeks of rHuEpo/placebo treatment, and after a 3 weeks washout period.

• EPO (Darbepoietin alpha) (~50IU/kg)/placebo (saline) is administered s.c. twice a week for the first 3 weeks at a dosage of 2x40 μg, and once a week from week 4-10 at a dosage of 20 μg.

• The endurance training will consist of 1 to 1.5 hours of cycling at 65-80% of VO_2_max, 3 times weekly. This was previously shown to cause an increase in maximum oxygen uptake (Midgley et al., 2006).


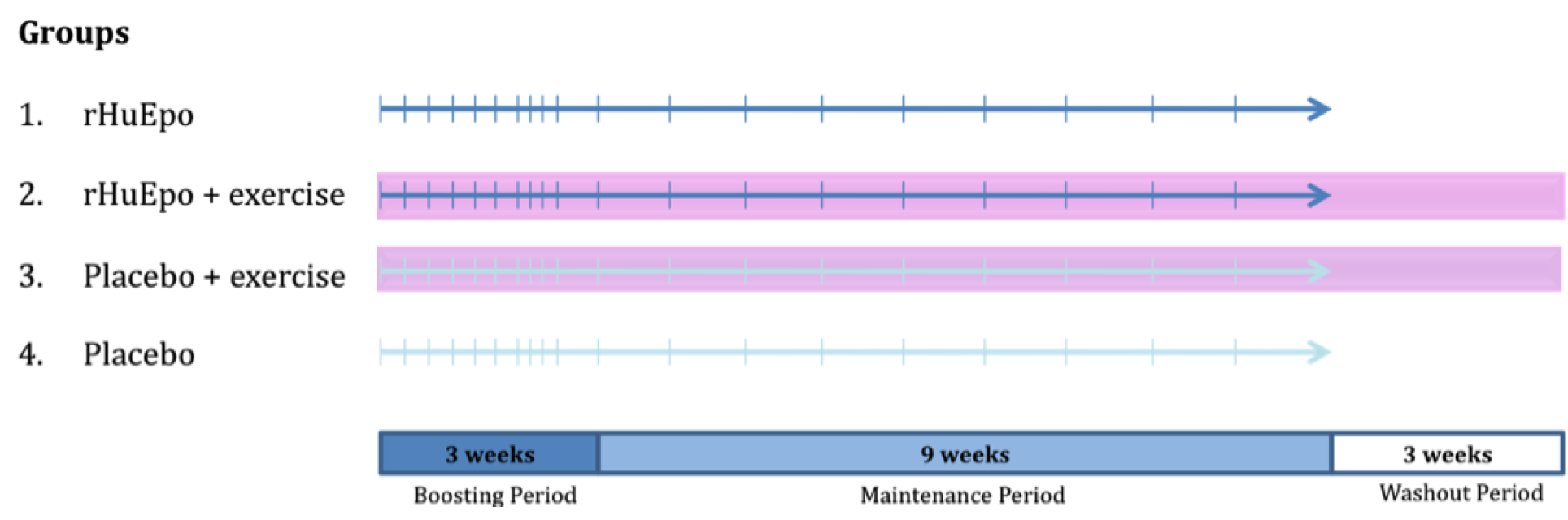


**Methods:**

• Metabolic profile, hyperinsulineamic eugluceamic clamp, muscle and fat biopsies, blood tests, maximum oxygen uptake, DEXA scan

**Primary end-points:** Proteomics analysis of serum

**Secondary end-points:** Substrate metabolism, fiber typing, and activation of satellite cells

**SUBJECTS**

A total of 40 healthy young men will be included in the present study. These will be recruited through the web page www.forsøgsperson.dk and various notices on educational institutions in Aarhus (see attached notice).

Because the effect of rHuEpo on some of the required parameters have not previously been elucidated, it has not been possible to make a power calculation. However, from similar studies in our laboratory, it is estimated that approximately 10 subjects in each group is enough to reach statistical power. Furthermore, in a previous study on a group of 8 subjects it was possible to detect significant changes in the proteome 16 days after treatment with rHuEpo (Christensen et al., 2010).

**Inclusion Criteria:**

• Written consent before study start

• Adult, healthy men

• Age >18 years and <35 years

• Normal weight (BMI: 18-27)

• Untrained (<2 hours of physical activity per week)

• Non smoking

**Exclusion Criteria:**

• Chronic disease

• Blood pressure above 135/85

• Hematocrit above 45%

**Participants will be excluded from the study if:**

• They want to end their participation.

• The investigator finds that the participant fails to conduct the experimental procedures or because of security reasons.

• Participants develop severe or intolerable side effects. If so, the experiment for each participant will be stopped and the investigator will refer to appropriate treatment. Hereafter, it is considered whether the trial must be terminated.

The participant may withdraw from the trial at any time. Data obtained from discontinued subjects will be used, if possible - it will depend on the duration until termination of participation. The data it is possible to analyze will be used.

**METHODS**

**Medical examination:** Thorough objective medical examination involving anamneses, measurement of blood pressure, waist circumference, weight, height, ECG, and routine blood tests. Routine blood tests will be obtained before starting the experiment, in order to ensure that the participants are healthy. The following will be measured; Hemoglobin, erythrocytes, iron, transferrin, ferritin, reticulocytes, haptoglobin, albumin, alkaline phosphatase bilirubin, LDH, plasma hemoglobin, ALT, potassium, sodium, creatininium, leukocytes, platelets, CRP, HbA1c, cholesterol, TSH, GFR.

**Maximal oxygen uptake:** Maximal oxygen uptake will be measured at least one week before the first metabolic day, on the same day as the screening blood tests are collected and the medical examination are carried out. The subjects will be re-tested again halfway through the training period and at the end of the study, the day after the final metabolic day. The subjects will be tested on an ergometer bicycle by indirect calorimetric. The aim is to achieve maximal oxygen uptake after approx. 5-7 min.

**DEXA scan:** Body composition will be measured by dual-energy X-ray absorptiometry (DEXA) on a Hologic Discovery prior to treatment and at the end of the treatment period.

**Metabolic profile:**

• **Hyperinsulineamic euglyceamic clamp:** Intravenous infusion of rapid-acting insulin (Actrapid, Novo Nordisk A/S, DK) 0.6 mU/kg TBW/min, while continuous intravenous infusion of a 20% glucose solution is adjusted in order to keep plasma glucose ~ 5 mM. Measuring blood glucose every 5-10 min and corresponding adjusting the glucose infusion rate ensure the level of glucose. The amount of infused glucose (M-value) is a measure of insulin sensitivity.

• **Carbamidtracer:** A bolus of ^13^C-urea (390.6 mg) followed by constant infusion of ^13^C-urea (42 mg/hr) for 4 hours.

• **Glukosetracer:** A bolus ^3^H_3_-glucose (20 μCi) and followed by constant infusion (0.20 μC/min) for 6 hours.

• **Palmitattracer:** FFA turnover is determined by Isotope dilution with 2 x 1 hour constant infusion of (9.10 -^3^H) palmitate (0.3 μCi/min giving a total of 18 x 2 μCi).

• **Amino acid tracers:** ^15^N-Tyrosine (0.3 mg/kg) and ^2^H_4_- Tyrosine (0.5 mg/kg) are given as a bolus. Then ^2^H_4_-Tyrosine (0.5 mg/kg/hr) is infused for 4 hours. ^15^N-Phenylalanine is given as a bolus (0.7 mg/kg) and then infused for 4 hours (0.7 mg/kg/hour).

• **Investigation of substrate metabolism in the forearm:** A plastic catheter is placed retrograde in a deep antecubital vein for collection of venous blood from the forearm muscle. On the opposite arm another catheter is placed antegrade in a vein on the back of the hand and in the antecubital vein. The hand is placed in a heated box whereby the blood is arterialized. Criteria for proper placement of the catheters are oxygen saturation below 70% for venous blood and more than 91% for arterialized blood. Before collecting blood, blood flow is measured with venous occlusion plethysmography. The blood flow to the hand is cut off with a cuff (250 mmHg) just before measuring blood flow and 1 minute before sampling from the vein. Arterial and venous blood is collected simultaneously.

• **Indirect calorimetric:** Indirect calorimetric (Oxycon Pro Care Fusion, Germany) is conduced 30 minutes prior to and at the end of the insulin clamp. This allows for calculations of total energy consumption, oxidative rates of glucose and fat, non-oxidative glucose turnover, and glucose output from the liver.

• **Biopsies:** Biopsies are taken one hour after the start of the metabolic profile and 30 minutes into the hyperinsulineamic clamp. *Fat Biopsies:* Subcutaneous fat is aspirated from the abdomen with a liposuction needle under local anesthesia and some of it is immediately frozen in liquid nitrogen. The remaining fat cells are purified by collagenase and stained with methylene blue for visualization of nukleii and cell membrane. Fat cell size is determined with a microscope and a camera is used to take pictures. *Muscle Biopsies:* A muscle biopsy is taken from the vastus lateralis with a Bergström needle. After 10-15 min. of local anesthesia (1% lidocaine), incision is made through skin and muscle fascia app. 15-20 cm above the knee. Muscle tissue is obtained and immediately frozen in liquid nitrogen or embedded in Tissue Tek and frozen. *Analyses:* Western Blot, PCR, immunohistochemical analysis, and intramuscular TG and glycogen.

• **Blood tests:** Blood samples are obtained every 20 minutes during the metabolic day. Total blood loss is around 320 ml. *Analysis:* Epo, total, free and bioactive IGF-I, IGFBP-1, glucose, insulin, C-peptide, free fatty acids, adiponectin, VLDL-TG, metabolites, glucagon, cortisol, catecholamines, albumin, phenylalanine enrichment, glucose specific activity, glycerol, lactate, ALT, alkaline phosphatase, bilirubin, pro-thrombin index, carbamid, ghrelin, and proteomics

• **VLDL studies:** These studies will be conducted before and after the 10 weeks intervention. 1 week in advance 60 ml of venous blood is sampled under sterile conditions. VLDL-TG will be insulated by ultracentrifugation (40000 g for 18 hours at 10 °C) and subsequent labeled with 20 μCi [1-^14^C] triolein. Sterility will be secured with cultivation before the sample is used for reinfusion. On the study day, the patient's own ex-vivo labeled VLDL-TG will be infused (autologous infusion) until steady state (4 hours). Blood samples will be obtained every 10 min over the last 30 min for determination of VLDL-TG SA and concentration.

• **VLDL-TG oxidation:** These studies will be performed before and after the 10 weeks intervention. VLDL-TG oxidation is measured by collecting expired air with hyamin-trapping for analysis of ^14^CO_2_, this is measured before the start of the metabolic day and 3 times during the last half an hour of the basal period.

- **MRI scanning:** MRI will be used to measure intrahepatic and intramyocellular lipid content. The liver and the tibialis anterior muscle will be scanned using a technique that combines MR imaging with localized proton MR spectroscopy with a voxel of 3x3x3cm, a 1.5T MR unit will be used together with a breath holding for 17 sec. Before entering the scanning room the subject will be asked about contra induced circumstances in relation to normal procedures for MRI. The subjects will be asked to remove any metal from pockets and remove jewelry. The subjects will be informed that MRI is a safe procedure without any risk of radiation. There will be a loud sound during the scanning, and the subjects will be given earplugs.

 
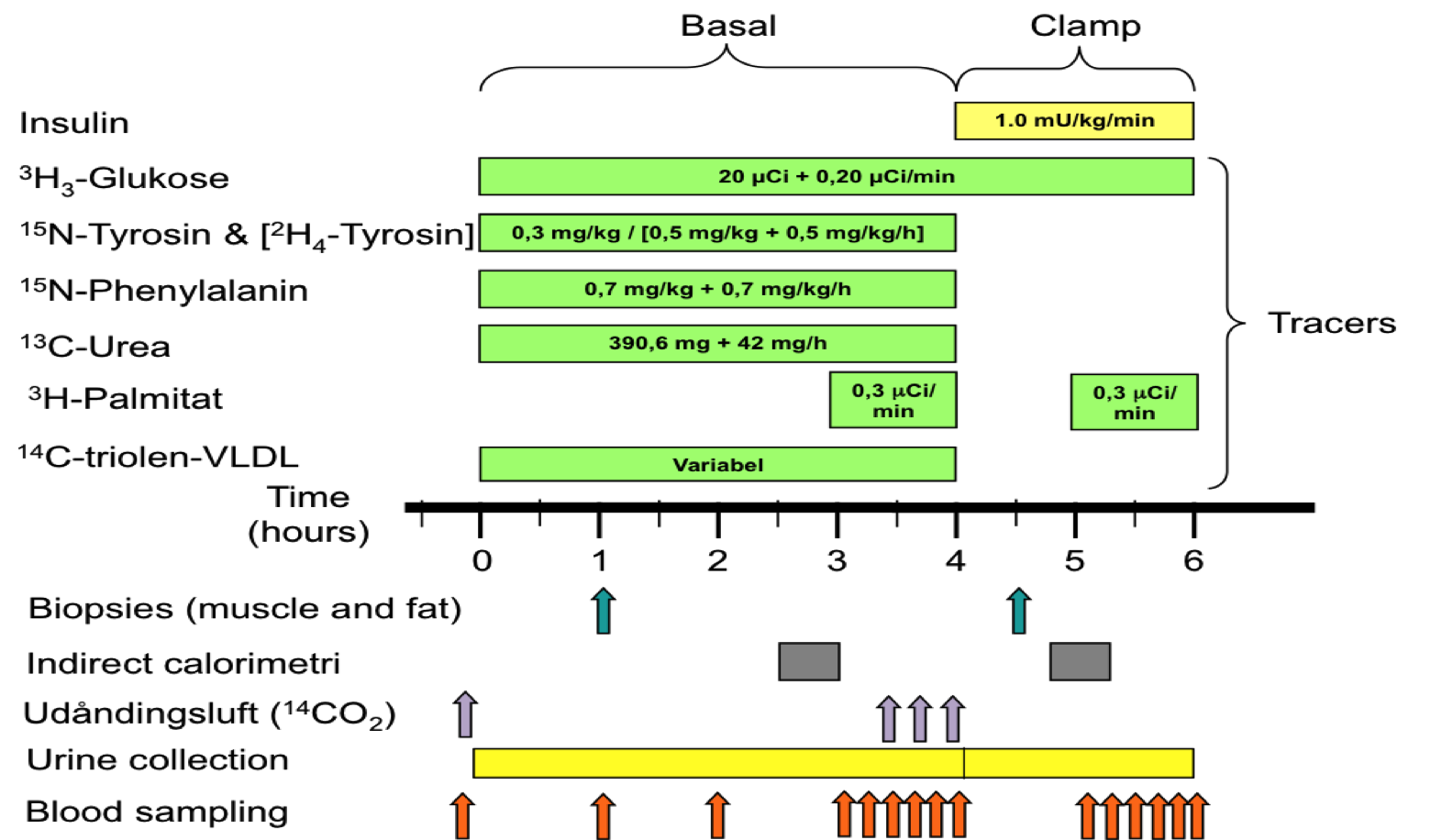


**Serum:** Serum will be collected before the study and on days 8, 15, 21, 36, 50, 64, 71, and 78 and 92 during the wash-out period. Hematological levels will be measured throughout the study, and treatment will be discontinued if the values are abnormal (Hematocrit >55%). The following will be measured; Hemoglobin, erythrocytes, iron, transferrin, ferritin, reticulocytes, haptoglobin, albumin, alkaline phosphatase, bilirubin, LDH, plasma hemoglobin, VLDL, FFA, ghrelin, and cholesterol. Proteomics analysis will be performed on blood samples taken before the study at baseline and on days 21, 71, and 92.

**Blood pressure measurements:** The subjects' blood pressure will be monitored throughout the study on the days when blood will be sampled. This is done to ensure that the treatment does not induce increased blood pressure.

Blood and tissue samples are used for analyses, and thereafter destroyed. A bio bank is therefore not created.

**SAFETY**

Before enrolling the participants in this trail a general medical examination will be performed.

Throughout the metabolic day a medical doctor and a laboratory technician will be present to make sure that the subjects are well and they will be able to measure vital parameters (blood pressure, pulse, blood sugar levels, and level of consciousness). Medical doctor Birgitte Nellemann will perform all invasive procedures, and clinically responsible for the project is Professor MD Jens Otto Lunde Jørgensen.

Acute treatment with Epo is not associated with serious side effects, but may be associated with transient flu-like symptoms, which will disappear within a few hours. Prolonged treatment may be associated with increased risk of thrombosis, hypertension and iron deficiency. A protocol similar to the Epo treatment regimen used here, has previously been approved by the Ethics Committee of Copenhagen and Frederiksberg (Lundby et al., 2007; Thomsen et al., 2007; Borno et al. 2010; Juel et al. 2007; Lundby et al., 2008). Here an increase in hematocrit from 45% to 49% (P <0.05) was found (Juel et al., 2007). The risk of thrombosis is greater if the subjects also have hypertension. Subjects will therefore be included only if their blood pressure is normal (<135/85) and they will be excluded if their hematocrit exceeds 55% (present doping limit is 50%, and the limit for venesection 55%). To avoid iron deficiency, all subjects will be treated with 100 mg iron orally daily from 1 week before the start of the experiment until the end.

When obtaining the biopsies there is a very small risk of infection or bleeding in the tissue. The subjects are instructed to contact the person responsible for the project by symptoms such as redness, swelling, warmth, and soreness. Additionally mild pain from the site of the biopsy can be experienced within the first few days after the procedure has been performed, equivalent to a muscle rupture. There is a minimal risk of damaging the small nerves in the skin, causing numbness in a small area on the skin. Sense of touch is often back after a shorter or rarely longer period of time. We also have had two cases of damage to a motor nerve in the muscle, which has resulted in local muscle atrophy without affecting muscle function.

When placing the intravenous catheters for blood collection and infusion there is a very small risk of infection and subjects are instructed to contact the investigator by redness and soreness corresponding to the injection sites. There is a small risk of hypoglycemia during and after the hyperinsuliniemic euglyceamic clamp. The risk is minimized by insuring a stable blood glucose level before the subject leaves the research laboratory. In addition, subjects are informed about symptoms of hypoglycemia and the subjects are given tablets with grape sugar for intake in the case of hypoglycemia.

Serious event or serious adverse event: an event that at any dose results in death, is life threatening, requires hospitalization, or prolongation of existing hospitalization, results in persistent or significant disability. If a serious event/adverse event appears this is reported to the Medicines Agency and the Ethics Committee. In case of a severe event the trail is immediately stopped and treatment commenced. The subject is followed until symptom free and/or the condition is stable. All adverse reactions/incidents will also be indicated in the final report. Complications will be recorded in connection with aforementioned medical examination and the subject will be treated at the research laboratory, since the trial is conducted in a highly specialized hospital.

The total blood loss at each experimental day is about 320 ml. There is 10 weeks between each experimental day, thus, this blood loss is not expected to induce symptoms. For comparison, an ordinary blood donation is approx. 500 ml. In addition, around 5 ml of blood will be collected on days 8, 15, 21, 36, 50, 64, 71 during the treatment period and at days 78 and 92 during the washout period, in order to monitor the hematological values. In addition, the participants will give 60 ml of blood for labeling of VLDL 1 week before the last experimental day. Blood loss in total throughout the study will be approximately 850 ml during a 4 months period.

**Radiation:**

The long-term risks by participating is related to radiation, and can be decomposed as follows: For measurement of glucose turnover radioactive labeled glucose will be infused corresponding to a total of 0.2 mSv and for the determination of FFA turnover the radiation will be 0.4 mSv for the palmitate tracer on each of the experimental days. The infused isotopes for measurement of urea and amino acid metabolism are stable and do not constitute any risk for the participant.

The total amount of radiation the subjects are exposed to on the experimental days is approximately 1.2 mSv., which is somewhat smaller than the radiation they will normally receive during a year (the background radiation is approximately 3 mSv/year) and less than the maximal dose limit (20 mSv per year for workers over 18 years). The DEXA scan is not associated with either pain or discomfort, but is associated with a radiation dose of 0.1 mSv. per scan, which corresponds to 1/30 of the annual background radiation in Denmark and 1/10 of the radiation dose a person would receive from an x-ray of the lungs. One can theoretically calculate the total additional risk of radiation to a 0.014% increase cancer risk during one's lifetime. An average Dane will therefore increase his risk from 25.000% to 25.014%.

**STATISTICS**

Group comparisons will be made with standard statistical methods (t-test and ANOVA or equivalent non-parametric tests). Within the groups, a paired t-test or equivalent non-parametric test will be used. A p-value below 0.05 is regarded as a significant result.

**SOURCE OF DATA**

From biopsies: Protein levels measured by Western blot and mRNA levels by PCR, fiber typing and activation of satellite cells measured by immunohistochemistry, fat cell size and activity assays for LPL.

From blood samples: Epo, total, free and bioactive IGF-I, IGFBP-1, glucose, insulin, c-peptide, free fatty acids, adiponectin, metabolites, glucagon, cortisol, catecholamines, albumin, enrichment of phenylalanine, glucose specific activity, glycerol, lactate, ALT, alkaline phosphatase, bilirubin, pro-thrombin index, carbamide, VLDL, triglyceride, VLDL-TG, proteomics analyses.

**QUALITY CONTROL AND QUALITY ASSURANCE**

The investigator and the research ethics committee or equivalent authority have access to verify all relevant data. The investigations follow GCP principles (Good Clinical Practice).

**ETHICAL CONSIDERATIONS**

**General**

It is hereby stated that the experiment will be conducted in accordance with the protocol and regulatory requirements. Information about participants is protected under the “Act on Processing of Personal Data” and the “Health Act”. Approval from the Ethics Committee of Central Region Denmark is required before the study is started. The study will be notified to the Danish Data Inspectorate, Clinical Trails Gov and if necessary to the Medicines Agency. The study will be conducted in accordance with the Helsinki Declaration II.

**Guidelines for written and oral information**

The oral and written information is provided in accordance with the Research Agency’s "Guidance on informing and obtaining consent from human subjects enrolled in biomedical research".

Potential volunteers may contact the investigator, and an appointment for oral information will be arranged. The subjects will receive the written information in advance of this meeting. The subjects will be informed about the possibility to have an accompanying person with them when the information is given. The oral information will be given by M.Sc. (Human Biology) Britt Christensen or doctor Birgitte Nellemann. The oral Information will be given in privacy.

There will be time to ask questions, to renounce knowledge about their own health and the pamphlet "Your rights as a subject in a biomedical research project" published by the Central Research Ethics Committee will be extradited. It will be emphasized that participation is voluntary and that participation may therefore be withdrawn at any time without affecting the doctor- patient relationship.

After the oral information is given, there will be at least 1 day of reflection before obtaining the written consent.

**Foreseeable risks and inconveniences**

Placement of the intravascular catheters is associated with mild pain, and there is a low risk of infection and hematoma at the injection site. Ordinary sterile techniques will be used.

The total blood loss at each of the experimental days is approximately 320 ml. There is 10 weeks between each of the experimental days, thus, blood loss is not expected to induce symptoms. For comparison, an ordinary blood donation is approximately 500 ml.

Biopsies are associated with some discomfort, but the procedure takes place under local anesthesia. All methods have been implemented in our experimental laboratory for a long time and it is judged to be absolutely acceptable in relation to the overall objective.

The total radiation dose the participants will receive is 1.2 mSv on each of the experimental days, which represents the same dose as received when having an X-ray of the pelvis. This radiation dose results in a 0.012% increased risk for contracting a cancer disease during one's lifetime.

DEXA scanning is not associated with either pain or discomfort, but the participants receives a radiation dose of 0.1 mSv per scan equivalent to 1/30 of the annual background radiation in Denmark and 1/10 of the radiation dose received when obtaining an X-ray of the lungs.

The total radiation dose (tracer + DEXA) increases the long-term risk of cancer with a total of 0.014%. The lifetime risk of cancer after participating in the study will rise from approx. 25% to approx. 25,014 %.

**Benefits of participation in the study**

The subjects may, upon completion of the project, get the results of own examinations and blood tests. The subjects who are randomly assigned to the training group will also engage in a 12-week supervised training regime. Besides this, it is estimated that there are no other immediate benefits for the individuals by participating in the study.

**Bio bank**

Blood and tissue samples will be used for analyses after the end of the study and possibly excess tissue will be destroyed thereafter. A bio bank is therefore not established. Blood samples will be sent to the U.S. where the proteomic analysis will be performed. Samples will be destroyed here if there is more left after the analyses are conducted. All the analyses are scheduled to be performed by the end of 2014.

**HANDLING AND ARCHIVING OF DATA**

In order to minimizing error when entering data, double entry and subsequent review will be performed. All data is stored in anonymous form. Data will be archived at the Department of Endocrinology and Internal Medicine, Aarhus University Hospital for 15 years after the study ends.

**FINANCE AND INSURANCE**

Funding: The project is funded by the Department of Endocrinology and Internal Medicine, MEA, and through a grant from the World Anti Doping Agency (WADA) (200,000 US$). Additionally, applications will be sent to private research funds. Investigator Britt Christensen is employed as a post doc at Aarhus University Hospital. The salary is paid by the Department of Endocrinology and Internal Medicine, MEA and WADA. PhD Student Birgitte Nellemann is paid by a stipend from Aarhus University.

Participants are covered in accordance with the law on complaints and compensation in accordance with the national health care services.

**PUBLICATION**

After termination of the study several English language articles will be prepared for publication in scientific per-reviewed journals. Both positive negative results will be published.

**COMPENSATION**

Participants in the study will receive a compensation of 5500 DDK if they are allocated to the training group and 4500 DDK if part of the control group. This covers any inconvenience associated with the experiment. Transportation to and from the two experimental days will be covered but the subjects themself will pay the remaining. The compensation is taxable. If a participant is withdrawn from the study he will receive compensation in accordance to the participation time.

**Identification of new serum markers for detection of abuse with erythropoietin**

Name: ..............................................................................................

**Consent Statement:**

"I hereby confirm that I have received orally as well as written information, and agrees to participate in the described scientific experiment.

I am informed that participation is voluntary and that I at any time and without justification may withdraw my commitment to participate, this without affecting any current or future treatment I may need."

Date: ........................

Signature: (study participant) ..................................................................

I hereby allow that my biological material can be stored in a research bio bank.

I want to get information on the experimental findings and any consequences for me.

Please circle: YES NO

Date: ..............................

Signature: (study participant) ..............................................................

Yours sincerely,

**M.Sc., Britt Christensen (project responsible), Medical doctor Birgitte Nellemann and Professor, Jens Otto Lunde Jørgensen (clinical responsible)**

**Medical Department of Endocrinology, MEA, Aarhus University Hospital**

______________________________________________________________

Reserved for the project responsible

"This is to certify that written and orally information has been given to the participant”:

Date: __________________ Signature: _____________________________

Layman description

**Identification of new serum markers for detection of abuse with erythropoietin**

Britt Christensen, Birgitte Nellemann, and Jens Otto Lunde Jørgensen

Department of Endocrinology and Internal Medicine, MEA and Medical

Research Laboratory, Aarhus University Hospital,

Norrebrogade 44, DK-8000 Aarhus C

Tel.: +45 89 49 20 35

**INVOLVED PARTNERS**

• Medical Research Laboratory

Aarhus University Hospital

Norrebrogade 44

8000 Aarhus C

Denmark

• Department of Endocrinology and Internal Medicine

Aarhus University Hospital

Norrebrogade 44

8000 Aarhus C

Denmark

• Institute of Sport Science

Aarhus University

Dalgas Avenue 4

8000 Aarhus C

Denmark

**TIMETABLE**

Estimated start: June 2011

Expected completion: August 2012

**LOCATION**

Medical Research Laboratory, Aarhus University Hospital.

Institute of Sports Science, University of Aarhus

**BACKGROUND**

Erythropoietin (Epo) is produced in the kidneys when the oxygen levels in the blood become too low. Epo stimulates the formation of new red blood cells in the bone marrow. Epo is used as a medicine for kidney and cancer diseases, leading to a lack of red blood cells.

Epo increases red blood cell production and hence the amount of oxygen that can be transported around the body. It is shown that prolonged use of synthetic Epo (rHuEpo) leads to an increase in the period of time a given physical work can be performed, therefore it will continue to be abused by athletes, especially in endurance sports. This happens despite the fact that rHuEpo was added to the anti-doping list already in 1990. It is therefore important to find a robust method to detect this abuse. Not only to ensure fair competition but also to reduce the abuse and the side effects that follow, e.g increased risk of blood clots.

The existing method for measuring the abuse of rHuEpo approved by the World Anti-Doping Agency (WADA) is based on differences in the sugar groups bound to rHuEpo and Epo produced in the body, respectively. Whether this approach will be effective against new synthetic Epo products are still unknown. Future Epo tests should therefore be based on biological markers, that changes when Epo levels rise. Various blood markers related to the amount of red blood cells have already been investigated, but these methods have proven to be very conservative, since the boundaries of the normal range is very wide in order to avoid false-positive results.

Proteomics is a method by which you can look at all the proteins in blood at the same time. Even proteins that have changed very little can be distinguished. The main objective of this project is to investigate the effect of prolonged treatment with rHuEpo on changes in blood proteins in healthy young men. We have in a previous study with 16 days of treatment with rHuEpo shown changes in blood proteins. It is hoped that some of these proteins can be used as marker for Epo abuse in the future. We furthermore want to explore the effect of physical activity on these proteins, since athletes will always be physically active. The effect of physical activity itself is therefore important to elucidate, if these proteins should be used in a future anti-doping test.

Epo receptors are found in many different types of tissues other than the bone marrow, for example, different cells in the brain, cancer cells, cells in the gut, and especially muscle tissue. The effects of Epo in these tissues are not fully elucidated.

We also want to investigate the effects of rHuEpo on fat and muscle tissue, and possible effects on the turnover of fat, sugar, and protein.

**PURPOSE**

To identify new biomarkers in the blood that can be used to detect rHuEpo abuse. In addition, to examine the effect of 10 weeks of training and treatment with rHuEpo at a wide range of other parameters such as turnover of fat, sugar, and protein, and changes in the muscle tissue.

**STUDY PLAN**

40 healthy young men will be included in the present study they will be recruited through the homepage www.forsøgsperson.dk and various notices on Aarhus University.

**Inclusion Criteria:**

• Written consent before study entry

• Adult, healthy men

• Age >18 years and <35 years

• Normal weight (BMI: 18-27)

• Untrained (<2 hours of physical activity per week)

• Non smoking

**Exclusion criteria:**

• Chronic disease

• Blood pressure above 135/85

• Hematocrit above 45%

**Design:**

• The subjects will be randomly allocated to one of the following 4 groups (10 persons in each group)

o Placebo treatment

o Placebo treatment + endurance training

o rHuEpo treatment

o rHuEpo treatment + endurance training

• Subjects are examined before and after 10 weeks rHuEpo/placebo treatment and again after a 3 weeks washout period.

• EPO (~50IU/kg)/placebo (saline) is administered using a small syringe under the skin twice a week for the first 3 weeks at a dosage of 2x 40μg, and once a week from week 4-12 at a dosage of 20μg.

• The endurance training will consist of 1 to 1.5 hours of cycling at 65-80% of maximum oxygen uptake, 3 times weekly.

 
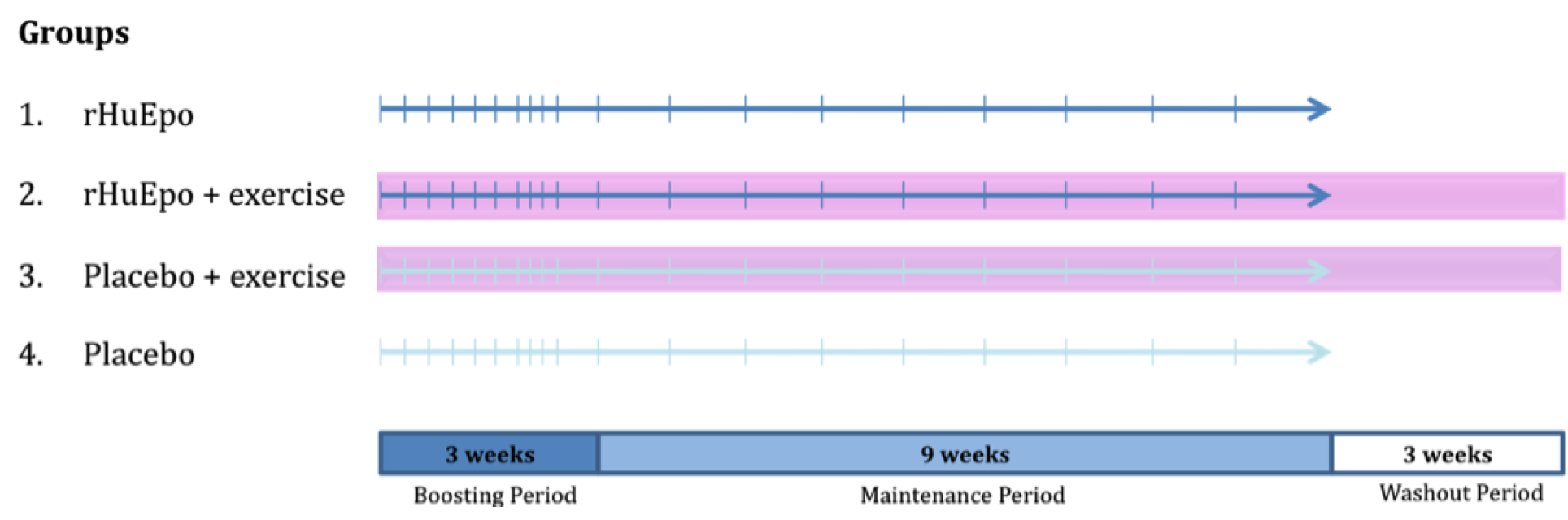


**Methods:**

• Fat, sugar, and protein metabolism, muscle and fat biopsies, blood samples, maximum oxygen uptake, DEXA scan, MRI scan

**Primary end points:** Proteomics analysis of blood samples

**Secondary end points:** The turnover of fat, sugar, and protein, changes in the muscle tissue

**Participants will be excluded from the study if:**

• They want to end their participation.

• The investigator finds that the participant fails to conduct the experimental procedures or because of security reasons.

• Participants develop severe or intolerable side effects. If so, the experiment for each participant will be stopped and investigator will arrange for appropriate follow-up treatment.

The participant may withdraw from the study at any time. Data obtained from discontinued subjects will be used, as far as possible - it will depend on the duration until termination of participation. The data it is possible to analyze will be used.

**METHODS**

**Medical examination:** Thorough objective medical examination will be made before the participants are enrolled in the study together with routine blood tests, a DEXA scan, and measurement of maximal oxygen uptake.

**Metabolic profile:** The experimental days take approx. 8 hours and will be conducted before treatment and again after 10 weeks of treatment. The subject should be fasting and lying in a bed.

o **Investigation of substrate metabolism in the forearm:** 3 thin plastic catheters are placed in 3 different veins for injection of various substances and to collect blood samples from. This allows for measurements of sugar, fat, and protein metabolism.

o **Biopsies:** Fat Biopsy: Fat biopsies are taken from underneath the skin on the stomach during local anesthesia. Muscle Biopsy: A muscle biopsy is taken from the thigh muscle during local anesthesia.

o **Blood tests:** Blood is sampled every 20 minutes during the experimental day. Total blood loss is approx. 320 ml.

o **Hyperinsulineamic euglyceamic clamp:** After approx. 4 hours we inject insulin for 2 hours to measure insulin sensitivity, while keeping the blood sugar levels constant.

o **Indirect calorimetric:** The expiration air is analyzed 30 min before and at the end of the insulin stimulation test. This allows us to calculate the total energy consumption, as well as sugar, protein, and fat turnover.

- **MR scanning**: MR will be used to access the lipid content in different tissues and organs. The subject will be lying down and be placed in the scanner, which is formed as a tube. The subjects will be informed about the MR scan, that it is a safe technique that does not induce any radiation. The will be a loud sound during the scanning and the subjects are offered earplugs.

**Blood tests:** Blood will be sampled before starting the treatment and on days 8, 15, 21, 36, 50, 64, 71 during the treatment, and on days 78 and 92 during the washout period. On these days, blood composition will be analyzed and if the values are abnormal, treatment will be discontinued. Proteomics analysis of blood samples will be performed on samples taken before the start of the study and on day 21, 71 and 92

**Blood pressure measurements:** Blood pressure will be measured on the same days as blood is collected.

Blood and tissue samples will be used for analyses immediately after termination of the study and hereafter destroyed. Thus, a bio bank is not created.

**SAFETY**

Before enrolling the participants in this trail a general medical examination will be performed.

Throughout the metabolic day a medical doctor and a laboratory technician will be present to make sure that the subjects are well and they will be able to measure vital parameters (blood pressure, pulse, blood sugar levels and level of consciousness).

Acute treatment with Epo is not associated with serious side effects, but may be associated with transient flu-like symptoms, which disappear within a few hours. Prolonged treatment may be associated with increased risk of thrombosis, hypertension, and iron deficiency. A protocol similar to the Epo treatment regimen used here has previously been approved. Here an increase in hematocrit from 45% to 49% (Juel et al., 2007) was found. The risk of thrombosis is greater if the subjects also have hypertension.

The subjects will therefore only be included if their blood pressure is normal (<135/85) and they will be excluded if their hematocrit exceeds 55% (the current doping limit is 50% and the limit for venosection is 55%). To avoid iron deficiency, all participants will be treated with 100 mg iron tablets daily from 1 week before starting the treatment until the trial is completed.

When obtaining the biopsies there is a very small risk of infection or bleeding in the tissue. The subject is instructed to report back to the investigators if they experience symptoms like redness, swelling, warmth, or soreness. Additionally mild pain from the site of the biopsy can be experienced within the first few days after the procedure has been performed. There is a minimal risk of damaging the small nerves in the skin, causing numbness in a small area on the skin. Sense of touch is often back after a shorter or rarely longer period of time. We also have had two cases of damage to a nerve in the muscle, which has resulted in loss of local muscle tissue but without any effect on muscle function.

When placing the thin plastic catheters for blood collection and infusion there is a very small risk of inflammation and the subjects are instructed to report back to the investigators if they experience redness and soreness corresponding to the injection sites.

There is a small risk of developing low blood sugar during and after the insulin stimulation test. Ensuring that blood glucose levels are normalized before the subjects are sent home minimizes this risk. In addition, subjects are informed about the symptoms of low blood sugar and the subjects are given tablets with grape sugar for intake in this case.

**Serious event or serious adverse event:** an event that at any dose results in death, is life threatening, requires hospitalization or prolongation of existing hospitalization, or results in persistent or significant disability. If a serious event/adverse events appear this is reported to the Medicines Agency and the Ethics Committee. In cases of a severe event the trail is immediately stopped and treatment commenced. The subject is followed until symptom free and/or the condition is stable. All adverse reactions/incidents will also be indicated in the final report. Complications will be recorded in connection with the aforementioned medical examination and the subject treated at the research laboratory, since the trial is conducted in a highly specialized hospital.

The total blood loss at each of the experimental days is approx. 320 ml. There is 10 weeks between each experimental day, thus this blood loss is not expected to cause any symptoms. For comparison, an ordinary blood donation is approx. 500 ml. In addition, the participants will give 60 ml blood for labeling of VLDL 1 week prior to the last experimental day. Furthermore, approx. 5 ml blood is collected on the following days 8, 15, 21, 36, 50, 71, 78 and 92 in order to monitor blood values.

**Radiation:**

The total radiation exposure is approx. 1.4 mSv., which is somewhat smaller than the radiation a person would normally receive during a year (Background radiation is approx. 3 mSv/year) and less than the dose limit (20 mSv per year for workers over 18 years). One can theoretically calculate the extra risk for developing cancer to 0.014% during one's lifetime. An average Dane will therefore increase his risk from 25.000% to 25.014%.

**STATISTICS**

Standard statistical methods will be used.

**ACCESS TO THE SOURCE OF DATA**

The investigator and the research ethics committee or equivalent authority have access to all relevant data.

**ETHICAL CONSIDERATIONS**

**General**

It is hereby stated that the experiment will be conducted in accordance with the protocol and regulatory requirements. Approval from the Research Ethics Committee of Central Region Denmark is required before initiating the project. The study will be notified to the Danish Data Inspectorate, Clinical Trials Gov, and if necessary the Medicines Agency. The study will be conducted in accordance with the Helsinki Declaration II.

**Guidelines for written and oral information**

The oral and written information will be provided in accordance with the Research Agency’s “Guidance on information and obtaining consent from human subjects enrolled in biomedical research.

Potential volunteers may contact the investigator, and an appointment for for verbal information will be arranged. The subjects will receive the written information before this meeting. The subjects will be informed about the possibility to have an accompanying person with them when the information is given. The oral information will be given by M.Sc. (Human Biology) Britt Christensen or medical doctor Birgitte Nellemann. The oral Information will be given in privacy.

There will be time to ask questions, to renounce knowledge about their own health and the pamphlet "Your rights as a subject in a biomedical research project" published by the Central Research Ethics Committee will be extradited. It will be emphasized that participation is voluntary and that participation can be withdrawn at any time without affecting the doctor- patient relationship.

After the oral information is given, there will be at least 1 day of reflection before obtaining the written consent.

**Foreseeable risks and inconveniences**

Insertion of the catheters in the veins is associated with mild pain, and there is a low risk of infection and hematoma at the injection site. Ordinary sterile techniques will be used.

The total blood loss at each of the experimental days is approx. 320 ml. There is 10 weeks between each experimental day, thus blood loss is not expected to cause any symptoms. For comparison, an ordinary blood donation is approx. 500 ml.

Muscle sampling is associated with some discomfort, but takes place under local anesthesia. All methods have been implemented in our experimental laboratory for years and are judged to be absolutely acceptable in relation to the overall objective.

The total radiation dose the participants will receive during the experiment is 1.4 mSv, which corresponds to less than an X-ray of the pelvis. This radiation dose results in a 0.014% increase in the risk for contracting a cancer disorder during one's lifetime.

**Benefits of participation in the study**

The subjects may, upon completion of the project, get the results of own examinations and blood tests. The subjects who are randomly assigned to the training group will also engage in a 12-week supervised training program. Besides this, it is estimated that there are no other immediate benefits from participating in the study.

**Bio bank**

Blood and tissue samples will be used for analyses immediately after completion of the study and the remaining tissue will be destroyed thereafter. Thus, a bio bank is no created. Blood tests will be sent to the U.S. where the proteomics analysis will be performed. Samples will be destroyed there if blood is left over after all the tests are finished.

**HANDLING AND ARCHIVING OF DATA**

In order to minimize error when entering data, double entry and subsequent review will be performed. All data is stored in anonymous form. Data will be archived at the Department of Endocrinology and Internal Medicine, Aarhus University Hospital, for 15 years after the study ends.

**FINANCE AND INSURANCE**

Funding: The project is funded by the Department of Endocrinology and Internal Medicine and through a grant from the World anti-doping agency (WADA) (200,000 US$). Additionally, applications will be sent to private research funds. Investigator Britt Christensen is employed as a post doc at Aarhus University Hospital. The salary is paid by the Department of Endocrinology and Internal Medicine and WADA. PhD Student Birgitte Nellemann is paid by a stipend from Aarhus University.

Participants are covered in accordance with the law on complaints and compensation in accordance with the national health care services.

**COMPENSATION**

Participants in the study will receive a compensation of 5500 DKK if they are allocated to the training group and 4500 DKK if part of the control group. This covers any inconvenience associated with the experiment. Transportation to and from the 2 experimental days will be covered but the subjects themselves will pay the remaining. The compensation is taxable. If a participant is withdrawn from the study he will receive compensation in accordance to the participation time.

**PUBLICATIONS**

After termination of the study several English language articles will be prepared for publication in scientific per-reviewed journals. Both positive and negative results will be published.

Participant Information

**Identification of new serum markers for detection of abuse with erythropoietin**

You are hereby invited to be part of a scientific project.

**PURPOSE**

To identify new biomarkers in the blood that can be used to detect abuse of Erythropoietin (Epo). Furthermore, to investigate the effect of 10 weeks of training and treatment with Epo on a variety of other parameters such as turnover of fat, sugar, and protein, and changes in the muscle and fat tissue.

**Epo**

Epo is produced in the kidneys when the oxygen pressure in the blood becomes too low. Epo increases the formation of new red blood cells in the bone marrow. Synthetic Epo (rHuEpo) is used to treat patients with low levels of red blood cells. Acute treatment with Epo is not associated with serious side effects, but may be associated with transient flu-like symptoms, which disappear within a few hours. Prolonged treatment may be associated with increased risk of thrombosis, hypertension, and iron deficiency. Blood composition and blood pressure will be monitored throughout the study, and iron supplements given in order to avoid iron deficiency.

Epo increases red blood cell production and hence the amount of oxygen that can be transported around the body. The same amount of physical work can be performed for a longer period after prolonged use of rHuEpo, therefore it will continue to be abused by athletes, especially in endurance sports. This despite the fact that rHuEpo was added to the anti-doping list already in 1990. It is therefore important to find a sensitive method to measure this abuse. Not only to ensure a fair competition but also to reduce the abuse and thus the side effects resulting from this abuse. In this study we want to detect new markers in the blood that can be used in a future anti-doping test.

Epo receptors are found in a variety of different types of tissues other than the bone marrow, eg. different cells in the brain, cancer cells, cells in the intestine, and especially muscle tissue, but the effect of Epo in these tissues still need to be fully elucidated.

Secondarily, we want to investigate the effects Epo on fat and muscle tissue, and possible effects on the turnover of fat, sugar, and protein. We have in a previous acute study found that Epo might induce changes in fat metabolism.

**STUDY DESIGN**

**You are:**

• Healthy

• 18-35 years

• Normal weight (BMI: 18-27)

• Untrained (<2 hours per week)

• Non smoking

• Normal blood pressure

**Study program:**

• Screening: Information about the study, a general medical examination involving measurement of blood pressure, waist size, weight, height, heart diagram, routine blood test, and measurement of body composition by a DEXA scan, and finally a maximum oxygen uptake test on a bicycle. Duration approx. 3 hours.

• A total of 2 full experimental days lasting approx. 8 hours before and after the 10-week intervention. In addition, a MR scanning before and after the 10 weeks.

• Blood will be sampled continuously during the 10-week intervention and 3 weeks thereafter (a total of 13 weeks).

• You will be randomly allocated to receive either rHuEpo or placebo (saline), which will be injected under the skin by one of the investigators. You will not know whether you are receiving rHuEpo or saline before the experiment is completed. You have to meet on the ward for treatment (perhaps in connection with the exercise) twice times a week for the first 3 weeks, and once a week for the last 7 weeks. On some of these days a blood sample will be drawn.

• Furthermore, you will also be randomly allocated to either a control or a training group. The training group will consist of 1 to 1.5 hours of cycling 3 times a week. The training will take place at the Department of Sport Science, Dalgas Avenue, Aarhus.

**The experimental day:**

• The experimental day lasts approx. 8 hours. You have to arrive at the medical research laboratory at 22 pm the day before the experimental day and stay overnight, and will on the experimental day be fasting and lying in a bed.

o Substrate metabolism in the forearm: 3 thin plastic catheters are inserted in 3 different veins for injection of various substances and blood sampling. This allows us to measure the metabolism of fat, sugar, and protein.

o Biopsy: After 1 and 5 hours a fat and muscle sample is obtained. *Fat Biopsy:* fat is obtained from beneath the skin on the abdomen under local anesthesia. *Muscle Biopsy:* A muscle sample is obtained from the thigh muscle under local anesthesia and can then feel like a "muscle rupture".

o Blood tests: Blood will be sampled approx. every 20 minutes throughout the experimental day. Total blood loss is approx. 320 ml.

o Insulin sensitivity: Insulin is responsible for the transport of sugar into the muscles. After approx. 4 hours we inject insulin for 2 hours in order to measure insulin sensitivity, while keeping the blood sugar levels constant.

o Indirect calorimetry: The expiration air is analyzed 30 min. before and at the end of the insulin stimulation test. This gives us the opportunity to calculate the total energy consumption, and the metabolism of sugar, protein, and fat.

**Participants will be excluded from the study if:**

• You want to withdraw from the study.

• The investigator finds that you fail to conduct the experimental procedures or because of security reasons.

• If you develop severe or intolerable side effects. In this case the trial will be stopped and the investigator will refer to appropriate treatment, and it is considered whether the trial must be terminated.

**RISKS**

Insertion of the catheters, fat and muscle biopsies, and injection of local anesthesia may be associated with mild discomfort and a small risk of development of hematoma and inflammation at the injection site. If you experience signs of inflammation (redness, warmth, soreness, and swelling) please contact the investigators (in daytime) or the emergency room (in the evening and night).

Muscle biopsies are associated by mild pain the days after the procedure, corresponding to a muscle rupture. There is a small risk of damaging the small nerves in the skin, this will for some time cause local numbness, but the sense of feeling will return. Furthermore, we have seen two cases of damage to a nerve in the muscle, which has resulted in local loss of muscle mass, but without any effect on muscle function. The total blood loss per experimental day will not exceed 320 ml.

A small amount of radioactive material will be used in the investigation of the metabolism of fat, sugar, and protein. The total radiation (1.2 mSv) is somewhat smaller than the background radiation you receive per year (3 mSv/year). The radiation dose is equivalent to less than an X-ray of the pelvis. In addition, each DEXA scan gives a radiation of 0.1 mSv. One can theoretically calculate the increased risk of contracting a cancer disease in the course of your lifetime to be increased by 0.014%. An average Dane will therefore increase his risk from 25.000% to 25.014 %.

The MRI scans does not induce any radiation. MRI scan can be problematic for subjects with claustrophobia, the scan is conducted while lying down inside a tube that will be open in both ends. During the examination there will be a loud sound, but you will be offered earplugs.

All the experimental methods have been used routinely in our research laboratory for several years.

**INFORMATION**

You will be informed both orally and written about the study purpose and objectives, and at will be clear that you at any time can withdraw from the experiment without justification, and this will not affect your treatment or doctor-patient relationship in the future.

You have the right to bring an assessor to the oral information, to request for time for consideration, and to renounce knowledge of your own health. You will receive the pamphlet "Your rights as a subject in a biomedical research study" published by the Central Ethical Committee. The study will be stopped if any serious side effects or serious incidents occur. You will be further informed if information emerges that are relevant to your for your continued participation in the trial.

**THE AIM OF THE EXPERIMENT**

Today, we know the effects Epo has on the blood and oxygen transport in the blood, but the effect elsewhere in the body is very poorly understood. Epo is used as a doping agent especially in endurance sports, and it is our hope that we will find new markers that can be used in a future anti-doping test. In addition, we hope that the investigation can help us to increase our knowledge on the effect of Epo on skeletal muscle tissue, specifically we are interested in finding out whether Epo affects the muscles sensitivity to insulin, or affect the metabolism of sugar, fat, and protein.

There are no immediate benefits for you as a participant in the study. However, if you are allocated to the training group, you will receive 10 weeks' supervised training. You can get the results and answers to your own blood tests after completion of the study.

**ECONOMY**

For participation in the study you will receive a compensation of 5500 DDK, if you are allocated to the training group and 4500 DDK if you are part of the control group, this amount covers any disadvantage associated with the experiment. In addition, we cover the transportation costs associated with the two experimental days, the remaining cost of transport will be covered by your self. The compensation is taxable. If you withdraw from the trial before completion you will receive compensation according to the time you have participated.

The project is funded by the Department of Endocrinology and Internal Medicine, MEA and through a grant from the World Anti Doping Agency (WADA) (200,000 USD).

**APPROVAL**

The project is approved by the Ethics Committee of Central Region Denmark and declared to the Data Protection Agency.

**DATA**

The project is reported to the Data Protection Agency and complies with the guidelines. Data is kept strictly confidential and stored for 15 years after completion of project. Information and details about your health and private affairs is strictly confident. You have the opportunity to gain access to your own information. Employees from the GCP unit and the local research ethics committee has access to lists of volunteers and can verify procedures and/or data in the study.

**COMPENSATION**

For your information, you have access to and opportunity for compensation related to the law on “complaints and compensation” under the national health care services.

**CONTACTS**

The practical part of the project will be carried out by:

Britt Christensen

Phone: 89 49 20 35

Mobile: 31 19 48 19

Email: britt.christensen @ ki.au.dk

Birgitte Nellemann

Phone: 89 49 20 32

Mobile: 29 61 14 46

Email: bnso@svf.au.dk

Clinical responsible are:

Consultant Jens Otto Lunde Jørgensen

Mobile: 20 72 73 83

In case of complications (as described above), please call the consultant at the Department of Endocrinology and Internal Medicine: 89 49 33 33 code 2067

**Are you a healthy, untrained male age 18-35 years, and do you want to participate in a scientific experiment?**

The aim is to investigate the effect of Epo and training.

To participate:

- You must be a man and not exercising on a daily basis

- You must be of normal weight with BMI 18-27

- You must be 18-35 years

- Do not take any medicine

The study includes:

- Screening with a medical checkup, examination of body composition by DEXA scan,

exercise test, and blood tests.

- 2 experimental days of 7-8 hours duration separated by 3 months. On each of the

experimental days, 2 fat and 2 muscle biopsies will be taken, as well as a MRI scan.

- You will be randomized to:

o Either 3 months supervised bicycle training, 3 times a week at Institute of Sports Science

o Or 3 months where you can not change your activity level

- Furthermore, you will be randomized to:

o Either weekly injections of Epo

o Or weekly injections with saline

- During the 3 months you will have blood tests obtained regularly.

Anticipated schedule:

- The project starts in August 2011 and there will be inclusion of subjects in the period

from August to November. The trial proceeds thereafter 3 months from the first

experimental day.

- If you allocated to the training group, you have to be able to participate in training at

the Institute of Sport Science, 3 times a week for 3 months.

Before the examination you will receive written and oral information. The project is approved by the Ethics Committee of Central Region Denmark. The project is support by a grant from the World Anti-Doping Agency (WADA). If you are allocated to the training group you will receive a taxable compensation of 5500 DDK or equivalent to the duration of your participation. I you are part of the control group will receive a taxable compensation of 4500 DDK or equivalent to the duration of your participation.

The experimental days are carried out at:

Medical Research Laboratory

Aarhus University Hospital

Norrebrogade 44, Building 3b

8000 Aarhus C

Training takes place at:

Institute of Sports Science

Dalgas Avenue 4

8000 Aarhus C

For further information contact:

Britt Christensen Birgitte Nellemann

M.Sc, PhD MD, PhD student

Department of Endocrinology Department of Endocrinology

Aarhus Hospital, Aarhus Hospital

Norrebrogade 44, 2b Norrebrogade 44, 2b

8000 Århus C 8000 Århus C

Tel: +45 8949 2035 Tel: +45 8940 2032

E-mail: britt.christensen @ ki.au.dk E-mail: birgitte.nellemann @ ki.au.dk

Reference List

Barroso O, Mazzoni I, & Rabin O (2008). Hormone abuse in sports: the antidoping perspective. *Asian J Androl* **10**, 391-402.

Borno A, Aachmann-Andersen NJ, Munch-Andersen T, Hulston CJ, & Lundby C (2010). Screening for recombinant human erythropoietin using [Hb], reticulocytes, the OFF(hr score), OFF ( z score) and Hb ( z score): status of the Blood Passport. *Eur J Appl Physiol* **109**, 1003-1005.

Catlin DH, Fitch KD, & Ljungqvist A (2008). Medicine and science in the fight against doping in sport. *J Intern Med* **264**, 99-114.

Christensen B, Sackmann-Sala L, Cruz-Topete D, Jorgensen JO, Jessen N, Lundby C, & Kopchick JJ (2010). Novel serum biomarkers for erythropoietin use in humans: A proteomic approach. *J Appl Physiol*.

Gore CJ, Parisotto R, Ashenden MJ, Stray-Gundersen J, Sharpe K, Hopkins W, Emslie KR, Howe C, Trout GJ, Kazlauskas R, & Hahn AG (2003). Second-generation blood tests to detect erythropoietin abuse by athletes. *Haematologica* **88**, 333-344.

Hojman P, Gissel H, & Gehl J (2007). Sensitive and precise regulation of haemoglobin after gene transfer of erythropoietin to muscle tissue using electroporation. *Gene Ther* **14**, 950-959.

Juel C, Thomsen JJ, Rentsch RL, & Lundby C (2007). Effects of prolonged recombinant human erythropoietin administration on muscle membrane transport systems and metabolic marker enzymes. *Eur J Appl Physiol* **102**, 41-44.

Lundby C, Hellsten Y, Jensen MB, Munch AS, & Pilegaard H (2008). Erythropoietin receptor in human skeletal muscle and the effects of acute and long term injections with recombinant human erythropoietin on the skeletal muscle. *J Appl Physiol* **104**, 1154-1160.

Lundby C & Robach P (2009). Assessment of total haemoglobin mass: can it detect erythropoietin-induced blood manipulations? *Eur J Appl Physiol* **108**, 197-200.

Lundby C, Thomsen JJ, Boushel R, Koskolou M, Warberg J, Calbet JA, & Robach P (2007). Erythropoietin treatment elevates haemoglobin concentration by increasing red cell volume and depressing plasma volume. *J Physiol* **578**, 309-314.

Midgley AW, McNaughton LR, & Wilkinson M (2006). Is there an optimal training intensity for enhancing the maximal oxygen uptake of distance runners?: empirical research findings, current opinions, physiological rationale and practical recommendations. *Sports Med* **36**, 117-132.

Pascual JA, Belalcazar V, de BC, Gutierrez R, Llop E, & Segura J (2004). Recombinant erythropoietin and analogues: a challenge for doping control. *Ther Drug Monit* **26**, 175-179.

Thomsen JJ, Rentsch RL, Robach P, Calbet JA, Boushel R, Rasmussen P, Juel C, & Lundby C (2007). Prolonged administration of recombinant human erythropoietin increases submaximal performance more than maximal aerobic capacity. *Eur J Appl Physiol* **101**, 481-486.
